# CRISPR/Cas9-mediated viral interference in plants

**DOI:** 10.1186/s13059-015-0799-6

**Published:** 2015-11-11

**Authors:** Zahir Ali, Aala Abulfaraj, Ali Idris, Shakila Ali, Manal Tashkandi, Magdy M. Mahfouz

**Affiliations:** Laboratory for Genome Engineering, Center for Desert Agriculture & Division of Biological Sciences, 4700 King Abdullah University of Science and Technology, Thuwal, 23955-6900 Saudi Arabia

**Keywords:** Plant genome engineering, CRISPR/Cas9 system, Synthetic site-specific nucleases, Viral-mediated genome editing, Virus resistance, *Tomato yellow leaf curl virus*, *Tobacco rattle virus*

## Abstract

**Background:**

The CRISPR/Cas9 system provides bacteria and archaea with molecular immunity against invading phages and conjugative plasmids. Recently, CRISPR/Cas9 has been used for targeted genome editing in diverse eukaryotic species.

**Results:**

In this study, we investigate whether the CRISPR/Cas9 system could be used in plants to confer molecular immunity against DNA viruses. We deliver sgRNAs specific for coding and non-coding sequences of tomato yellow leaf curl virus (TYLCV) into *Nicotiana benthamiana* plants stably overexpressing the Cas9 endonuclease, and subsequently challenge these plants with TYLCV. Our data demonstrate that the CRISPR/Cas9 system targeted TYLCV for degradation and introduced mutations at the target sequences. All tested sgRNAs exhibit interference activity, but those targeting the stem-loop sequence within the TYLCV origin of replication in the intergenic region (IR) are the most effective. *N. benthamiana* plants expressing CRISPR/Cas9 exhibit delayed or reduced accumulation of viral DNA, abolishing or significantly attenuating symptoms of infection. Moreover, this system could simultaneously target multiple DNA viruses.

**Conclusions:**

These data establish the efficacy of the CRISPR/Cas9 system for viral interference in plants, thereby extending the utility of this technology and opening the possibility of producing plants resistant to multiple viral infections.

**Electronic supplementary material:**

The online version of this article (doi:10.1186/s13059-015-0799-6) contains supplementary material, which is available to authorized users.

## Background

In bacteria and archaea, the clustered regularly interspaced palindromic repeat (CRISPR)/ CRISPR-associated (Cas) 9 (CRISPR/Cas9) system confers molecular immunity against nucleic acids of invading conjugative plasmids or phages [1–6]. The CRISPR/Cas9 system has recently been harnessed in diverse eukaryotic species, including plants, for the purpose of targeted genome editing and regulation [7, 8]. The CRISPR/Cas9 molecular immunity system comprises the Cas9 endonuclease of *Streptococcus pyogenes* and a synthetic single guide RNA (sgRNA), which directs the Cas9 endonuclease to a target sequence complementary to the 20 nucleotides preceding the protospacer-associated motif (PAM) NGG, which is required for Cas9 activity [9, 10]. Thus, engineering of the system for a user-selected target requires only the addition of 20 nucleotides to the sgRNA molecule, allowing facile targeted genome editing and regulation. Furthermore, simultaneous targeting of several genomic loci (multiplexing) is feasible using multiple sgRNAs [7].

Geminiviruses, a large family of plant DNA viruses, cause severe crop losses and economic consequences that threaten food security worldwide, especially in underdeveloped regions like sub-Saharan Africa [11, 12]. Members of the *Geminiviridae* possess a twin icosahedral capsid structure that encapsidates a circular single-stranded DNA (ssDNA) genome between 2.3 and 3 kb in length. *Geminiviridae* viruses replicate their genome either by a rolling-circle amplification (RCA) mechanism via a double-stranded DNA (dsDNA) replicative form (similar to that of Φ174 phage), or by recombination-mediated replication [13]. Geminiviruses do not encode their own DNA polymerase, but instead reactivate S phase and the cellular machinery to facilitate replication of their own genome [14]. Upon infection of plant cells, the viral Rep protein binds to the origin of replication, thereby initiating viral replication in the nucleus [15]. Based on their genome organization, host range, insect vectors, and genome-wide pairwise sequence identity, members of *Geminiviridae* are classified into seven genera: *Begomovirus*, *Mastrevirus*, *Curtovirus*, *Becurtovirus*, *Eragrovirus*, *Turncurtovirus* and *Topocuvirus.* Members of the genus *Begomovirus*, which infect dicotyledonous plants via whitefly transmission vectors, include bipartite and monopartite viruses [16]. Bipartite viruses, such as African cassava mosaic virus and cabbage leaf curl virus, have genomes comprising two components (A and B) that differ in sequence (except for the common region, a 200–250-bp sequence that is nearly identical) [14]. The common region forms part of the highly conserved intergenic region (IR), which contains the origin of replication and promoter sequences for RNA polymerase II [17]. By contrast, monopartite viruses, such as tomato yellow leaf curl virus (TYLCV), have a single genomic component. A conserved nucleosome-free IR is present in the minichromosome of all geminiviruses [17, 18].

**Fig. 1:**
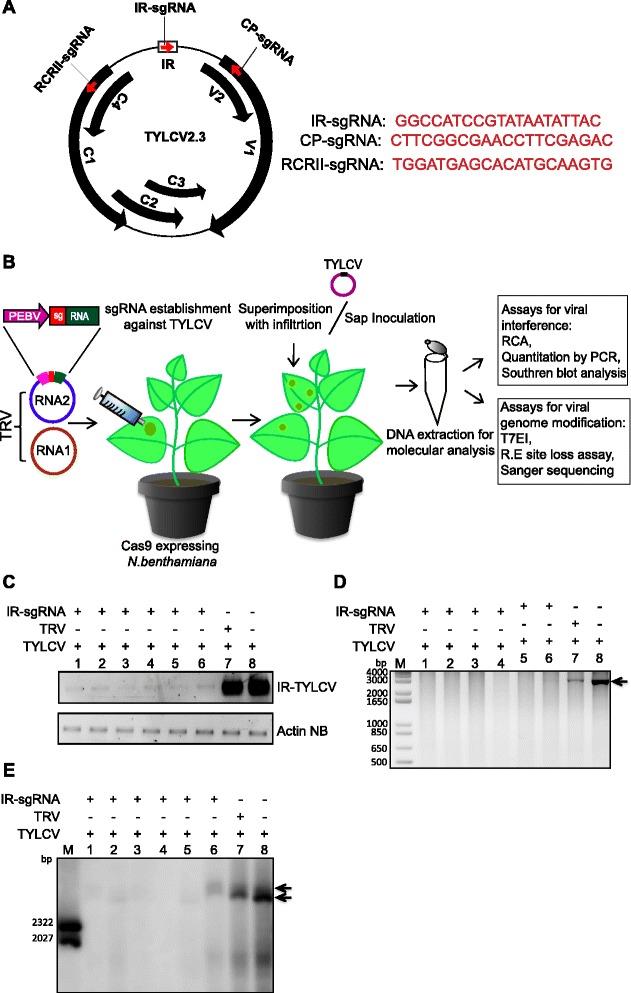
CRISPR/Cas9-mediated interference with accumulation of the TYLCV genome. **a** Genome organization of TYLCV. The six partially overlapping ORFs are represented by *black filled arrows*, and the IR is represented by an *open box*. The three CRISPR/Cas9 targets are represented by *red arrowheads*. The sequences of the three targets (IR, CP, and RCRII) are shown on the right. **b** Schematic representation of the experimental design. *Agrobacterium* containing engineered tobacco rattle virus (*TRV*) with sgRNA specific for the TYLCV genome was infiltrated into Cas9-expressing plants. TYLCV was subsequently infiltrated to Cas9OE plants harboring an established TRV infection. Samples were collected at 10–21 days post-infiltration (dpi) for molecular analysis. **c** Semi-quantitative PCR of TYLCV genomic DNA. TYLCV infiltration into Cas9OE plants harboring IR-sgRNA accumulated lower levels of TYLCV than plants infiltrated with the TRV empty vector. Actin genomic DNA from *N. benthamiana* was used for normalization. **d** Assay for rolling-circle amplification (*RCA*) of the TYLCV genome in plant extracts. Accumulation of TYLCV genomic DNA in plants harboring IR-sgRNA was lower than that in plants inoculated with TYLCV and the TRV empty vector. **e** Southern blot analysis of TYLCV genomic DNA accumulation in Cas9OE plants. TYLCV genomic DNA was detected with a DIG-labeled probe against a 560-bp sequence within the IR region. All six individual IR-sgRNA-harboring plants that were infiltrated with TYLCV showed lower accumulation of the TYLCV genome than plants inoculated with the TRV empty vector and TYLCV. *Arrowheads* in (**d**, **e**) indicate the expected size of the TYLCV genome. *DIG* Digoxigenin, *M* DNA size marker, *NB N. benthamiana*, *PEBV* pea early browning virus promoter, *RE* restriction enzyme

TYLCV, a member of the genus *Begomovirus*, causes widespread destruction of tomato crops worldwide [19]. TYLCV is a ssDNA virus, with a genome of approximately 2.7 kb [20, 21]. The genomic structure of TYLCV (Fig. 1a) consists of six bi-directionally organized, partially overlapping open reading frames (ORFs), with an IR containing the origin of replication [15]. Disease symptoms caused by TYLCV include chlorotic leaf margins; cupped, thick, and rubbery small leaves; significant fruit abscission; and overall stunting of plants [20]. Control and management of the disease caused by TYLCV have proven both challenging and expensive. Previous approaches to developing disease resistance focused on insecticides targeting the viral transmission vector, the silverleaf whitefly (*Bemicia tabaci*) [16]. Breeding for resistance has been equally challenging due to linkage between the resistance locus and genes associated with poor fruit quality [22, 23]. Several attempts have been made to engineer tomato plants that are resistant to TYLCV, including over-expressing the viral capsid protein (CP) and C4 protein or the non-coding IR sequences [24]; the latter approach is based on the idea that binding of the Rep protein to the origin of replication might interfere with viral replication. A synthetic zinc finger protein has been used to block the replication protein (Rep, C1) of beet severe curly top virus from binding to the origin of replication, resulting in virus resistance [25]. A similar approach was applied to TYLCV [26, 27]. Nonetheless, there is currently no effective means of controlling or managing TYLCV disease. Therefore, to increase the yield of crops infected by this and related viruses, it will be necessary to develop efficient technologies for conferring viral resistance [28].

Targeted DNA mutagenesis using site-specific endonucleases has been used against a variety of mammalian DNA viruses [29]. Very recently, the CRISPR/Cas9 system was used to target different mammalian viruses. For example, this technology was used to efficiently eradicate HIV proviral DNA from the host genome and prevent HIV infection [30]. It was also used to disrupt the hepatitis B virus, treat latent infection by Epstein-Barr virus, and engineer the large genome of the herpes simplex virus [31–34]. A recent report showed the feasibility of targeting a eukaryotic RNA virus using the CRISPR/Cas machinery [35]. In this work, we demonstrate the use of CRISPR/Cas9 for in planta viral interference against TYLCV. Both the TYLCV ORFs and the IR sequence could be targeted for cleavage and mutagenesis by the CRISPR/Cas9 system. Targeting of the TYLCV genome resulted in significant reduction or attenuation of disease symptoms. Further, the CRISPR/Cas9 system was able to target multiple virus sequences simultaneously. Therefore, it may be generally possible to use the CRISPR/Cas9 system to develop plants that are resistant to TYLCV and other DNA viruses.

## Results

### CRISPR/Cas9-mediated interference with TYLCV

In this study, we investigated whether the CRISPR/Cas9 system can be used in plants to impart molecular immunity against DNA viruses. To this end, we used our recently developed system for genome editing, which involves systemic delivery of sgRNA molecules via tobacco rattle virus (TRV) into *N. benthamiana* plants overexpressing Cas9 endonuclease (NB-Cas9OE) [36]. We designed sgRNAs specific for different TYLCV coding and non-coding sequences (Fig. 1a), and inserted them into the TRV RNA2 genome. Next, we delivered the sgRNAs via agroinfection of TRV into NB-Cas9OE plants. Seven days post-infiltration (dpi) with TRV, we challenged the NB-Cas9OE plants with an infectious TYLCV clone via agroinfection (Fig. 1b) [37]. Ten days later, we isolated total RNA and DNA from the NB-Cas9OE plant systemic leaves for various molecular studies. To determine TYLCV titer, we performed semi-quantitative PCR using primers encompassing the IR region (Table S1 in Additional file 1). The titer was lower in samples co-infiltrated with sgRNA targeting the IR region than in those infiltrated with TRV vector controls and TYLCV (Fig. 1c). TYLCV replicates through a RCA mechanism that exploits the plant machinery [38]. An RCA assay revealed that targeting of the IR via the CRISPR/Cas9 system prevented accumulation of the TYLCV genome (Fig. 1d). Because TYLCV is a ssDNA virus that is converted to dsDNA inside the plant cell nucleus, interference with TYLCV replication by targeting the IR within replicating viral dsDNA should significantly reduce the accumulation of both the ssDNA and dsDNA forms. To test for interference with TYLCV replication, we performed dot blot assays. The results showed that the titer of TYLCV in IR-sgRNA plants was lower than that of the TRV vector control (Figure S1 in Additional file 2). In addition, we validated our dot blot results by Southern blotting, which confirmed that targeting the IR of TYLCV prevented accumulation of both ssDNA and dsDNA (Fig. 1e).

We further confirmed our findings by using a different method for TYLCV inoculation, namely, the sap transmission method (Additional file 3). Sap from young leaves of TYLCV-infected wild-type *N. benthamiana* plants was directly applied to *N. benthamiana* Cas9OE plants 7 days after infection with TRV-sgRNA. DNA was extracted from the systemic leaves after 21 days of sap application and then subjected to different types of molecular analysis. Non-specific sgRNA (possessing no sequence similarity to the TYLCV genome; Supplementary sequence 9 in Additional file 4) rather than an empty TRV vector was used in the sap transmission experiments. The RCA results revealed a reduction in TYLCV genome accumulation in both samples treated with IR-sgRNA or CP-IR-sgRNA compared with samples treated with non-specific sgRNA or TYLCV alone (Figure S2a in Additional file 2). To confirm the RCA results, we next performed semi-quantitative PCR to amplify a 560-bp fragment encompassing the TYLCV IR. The results revealed lower amplification of TYLCV with specific sgRNAs than with controls (Figure S2b in Additional file 2), thereby confirming the RCA results. Both the RCA and semi-quantitative PCR assays are based on the amplification of available TYLCV genome. To further confirm these data, we next performed Southern blotting, which confirmed lower accumulation of TYLCV in the presence of specific sgRNAs than in the presence of controls (Figure S2c in Additional file 2).

### CRISPR/Cas9 mediates targeted cleavage of the TYLCV genome

**Fig. 2:**
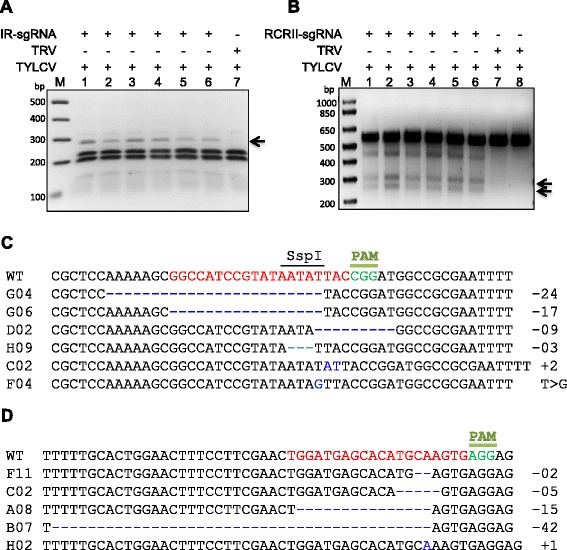
CRISPR/Cas9-mediated targeted cleavage of the TYLCV genome. **a** Mutation analysis using a restriction site loss assay. The TYLCV IR (560 bp) was analyzed for loss of the *Ssp*I recognition site at the targeted locus. The *arrow* indicates the presence of a 269-bp *Ssp*I-resistant DNA fragment only in samples harboring IR-sgRNA, but not in samples harboring the TRV empty vector. **b** T7EI assay for detecting indels in the RCRII domain of the TYLCV genome. The T7EI assay detected mutations only in RCRII PCR amplicons from plants infiltrated with TRV containing RCRII-sgRNA, but not in plants infiltrated with TRV empty vector. DNA fragments A and B were resolved on a 2 % agarose gel and stained with ethidium bromide. *Arrows* show the expected DNA fragments. **c** Alignment of reads from PCR amplicons encompassing the IR region, which were subjected to Sanger sequencing. **d** Alignment of reads from the PCR amplicons encompassing the RCRII motif, which were subjected to Sanger sequencing. The wild-type (*WT*) TYLCV sequences are shown at the top. The target sequence is shown in *red*, the *Ssp*I site is indicated by a *line*, and the protospacer-associated motif (*PAM*) is indicated in *green*. This is followed by the various indels, which are indicated by the numbers to the right of the sequence (−x indicates deletion of x nucleotides; +x indicates insertion of x nucleotides; and T > G indicates change of T to G). Arrows indicate the expected sizes of the cleavage products

We subsequently investigated whether the attenuated replication of TYLCV was indeed due to targeted cleavage or modification of the genome, rather than simply to interference with the replication machinery resulting from binding by the CRISPR/Cas9 complex. To this end, we employed T7EI and restriction site loss assays to confirm targeting and determine the efficiency of modifications within the selected sequences. The 20-nucleotide target sequence of the IR of the TYLCV contains a recognition sequence for *Ssp*I endonuclease at the predicted cleavage site 3 bp upstream of the PAM sequence. We isolated genomic DNA at 10 dpi with the TYLCV infectious clone and PCR amplified a 560-bp fragment encompassing the IR target sequence, which contains two additional *Ssp*I sites (Supplementary sequence 1 in Additional file 4). Complete *Ssp*I digestion of the wild-type sequence produced four fragments of 53, 92, 189, and 216 bp; targeted modification of the IR sequence and subsequent repair via non-homologous end joining eliminated the *Ssp*I site within the IR, generating a 269-bp *Ssp*I-resistant band. We observed the 269-bp band only in the IR-sgRNA samples, indicating successful targeted modification of the IR by the CRISPR/Cas9 system (Fig. 2a). To confirm the presence of indels, we cloned the 560-bp PCR amplicons into the pJET 2.1 cloning vector and performed Sanger sequencing. Alignment of the sequencing reads of 300 clones indicated that 42 % of the clones carried targeted modifications within the IR sequence (Fig. 2c; Table S2 in Additional file 1). Furthermore, to determine whether targeting ORFs could also mediate interference with TYLCV, we designed sgRNAs targeting the CP and RCRII motif of the Rep ORF. The T7EI assays and Sanger sequencing indicated that different ORFs could be targeted for modification to interfere with TYLCV accumulation (Fig. 2b, d; Figure S3 in Additional file 2; Table S2 in Additional file 1). We confirmed the results of the T7EI assays by performing RCA and Southern blotting assays (Figures S4 and S5 in Additional file 2). In nature, DNA viruses are transmitted by different means and vectors. Therefore, we wondered whether our system was capable of targeting the sap-transmitted TYLCV genome. DNA was extracted from sap-transmitted TYLCV and used to infect *N. benthamiana* Cas9OE plants expressing IR-sgRNA, CP-IR-sgRNA, or controls. The corresponding CP fragment (642 bp) from CP-IR-sgRNA and the IR fragment (560 bp) from CP-IR-sgRNA or IR-sgRNA were PCR amplified and subjected to *Bsm*BI (CP) and *Ssp*I (IR) recognition site loss assays. DNA fragments resistant to *Bsm*BI in CP amplicons and to *Ssp*I in IR amplicons were detected in CP-IR-sgRNA samples but not in controls (Figure S2d in Additional file 2). The corresponding *Ssp*I-resistant fragment was also observed in IR-sgRNA samples but not in controls (Figure S2e in Additional file 2).

### CRISPR/Cas9 system mediates specific and multiple targeting of viral genomes

**Fig. 3:**
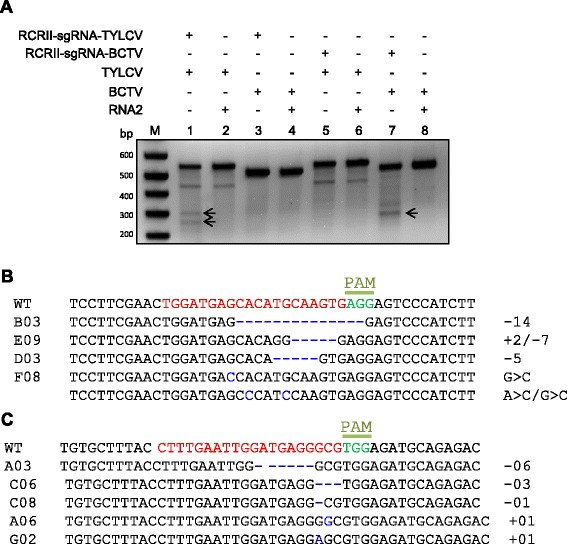
Specific targeting of different viral genomes. RCRII sgRNAs specific for TYLCV and BCTV sequences target only the TYLCV and BCTV genomes, respectively. **a** T7EI assays showing specific targeting of the TYLCV or BCTV genomes. **b** Alignment of Sanger sequenced reads from the TYLCV-targeted RCRII region. **c** Alignment of Sanger sequenced reads from the BCTV-targeted RCRII region. The various indels are indicated by the numbers to the right of the sequences (−x indicates deletion of x nucleotides; +x indicates insertion of x nucleotides; and X > Y indicates change of nucleotide X to nucleotide Y). Arrows indicate the expected sizes of the cleavage products. *WT* wild type

We next asked whether the CRISPR/Cas9 is capable of mediating specific interference with TYLCV. Notably, the RCRII motifs of the Rep ORFs of geminiviruses are conserved at the amino acid level but variable at the nucleotide level. To confirm that our RCRII-sgRNA targeted only the TYLCV RCRII region and interfered specifically with TYLCV genome replication, we co-infiltrated another monopartite geminivirus, beet curly top virus (BCTV) strain Worland (Supplementary sequence 7 in Additional file 4), along with the TYLCV-RCRII-sgRNA. We tested for modifications of the RCRII sequences of both TYLCV and BCTV using T7EI assays. The results confirmed that TYLCV-RCRII-sgRNA specifically targeted the TYLCV genome, but not the BCTV genome (Fig. 3a). We confirmed that BCTV-RCRII-sgRNA targeted the BCTV genome but not the TYLCV genome (Fig. 3a). Sanger sequencing data confirmed the results of T7EI assays with regard to specific targeting of each genome (Fig. 3b, c).

**Fig. 4:**
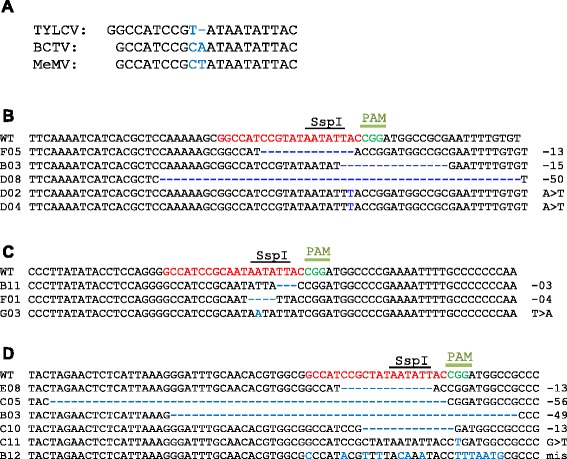
Targeting of different geminivirus genomes using a single sgRNA. A single IR-sgRNA was capable of targeting the TYLCV, BCTV, and MeMV genomes. **a** IR-sgRNA (*upper sequence*) identical to the TYLCV IR sequence but harboring mismatches with the BCTV and MeMV IR sequences (*blue*) was used to target all three viral genomes. **b**–**d** Alignment of Sanger sequenced reads from the IR-targeted region in the TYLCV, BCTV, and MeMV genomes showing the respective targeted modifications. The wild-type (*WT*) sequence is shown at the top (*red*) and the PAM is shown in *green*

Because the stem-loop sequence of the origin of replication in the IR is conserved in all geminiviruses, we investigated the possibility of targeting different viruses with a single sgRNA. We designed an IR-sgRNA that contains the invariant TAATATTAC sequence common to all geminiviruses (Fig. 4a) [39] and tested this IR-sgRNA against TYLCV and BCTV. Sanger sequencing confirmed the presence of indels and targeted modifications in both viruses (Fig. 4b, c; Table S2 in Additional file 1). Because monopartite and bipartite geminiviruses share the same conserved stem-loop sequence in the origin of replication within the IR (Fig. 4a), we next targeted a bipartite virus, the Merremia mosaic virus (MeMV) (Supplementary sequence 8 in Additional file 4) [40]. Sanger sequencing confirmed that IR-sgRNA specific to TYLCV but containing the invariant TAATATTAC sequence targeted a similar sequence in the MeMV genome (Fig. 4d; Table S2 in Additional file 1). Thus, a single sgRNA is capable of targeting multiple viruses.

### The CRISPR/Cas9 system attenuates or represses TYLCV symptoms

Interference with TYLCV replication by the CRISPR/Cas9 machinery is predicted to eliminate or reduce TYLCV symptoms, reminiscent of the CRISPR/Cas9 system’s originally evolved function in natural immunity of bacteria against phages. Accordingly, we assessed and evaluated TYLCV symptoms in NB-Cas9OE plants expressing sgRNAs against TYLCV coding and non-coding sequences. In these experiments, we challenged three groups of NB-Cas9OE plants, which expressed sgRNAs specific to the IR, CP, or Rep regions, with an infectious TYLCV clone. NB-Cas9OE plants expressing sgRNA targeting the IR exhibited significantly reduced TYLCV symptoms relative to the TRV vector control (Figure S6 in Additional file 2; Table S2 in Additional file 1). Moreover, NB-Cas9OE plants expressing sgRNA targeting the CP or Rep ORF also exhibited a reduction of TYLCV symptoms, but the magnitude of this reduction was smaller than that achieved by targeting the IR-sgRNA (Figure S7 in Additional file 2; Table S2 in Additional file 1). In a second set of experiments, we investigated whether targeting more than one sequence of the TYLCV genome would lead to greater reduction of TYLCV symptoms. To test this, we co-infiltrated a pair of TRV RNA2 genomes carrying sgRNAs targeting the CP region and IR. Targeting two different sequences did not have an additive effect on the reduction of TYLCV symptoms (Figure S8 in Additional file 2; Table S2 in Additional file 1).

Because co-infiltration of two RNA2 genomes carrying two different sgRNAs does not ensure the delivery of both sgRNAs into a single cell (and their subsequent activities against single molecules of the viral genome), we multiplexed our sgRNA delivery using the recently developed polycistronic tRNA–gRNA (PTG) system [41]. Subsequently, to determine the activity of both gRNAs in this system, we delivered a single RNA2 genome carrying both IR-sgRNA and CP-sgRNA. This TRV system was capable of expressing two sgRNAs that can target both IR and CP sequences. Restriction site loss assays (Figure S9 in Additional file 2) and Sanger sequencing confirmed the targeted modification of both IR and CP sequences (Figure S10 in Additional file 2). Furthermore, simultaneous targeting of two sequences by PTG-based expression led to greater reduction of virus titer and recovery of disease symptoms in NB-Cas9OE plants (Figure S11 in Additional file 2; Table S2 in Additional file 1). Next, we performed Southern blotting to confirm the absence or reduced accumulation of the TYLCV genome in IR-CP-sgRNA infiltrated plants relative to that in plants infiltrated with the vector control. The molecular analyses showed a significant reduction in viral genome levels when the IR and CP region were simultaneously targeted for cleavage. This confirmed the phenotypic data regarding the TYLCV symptoms (Figure S12 in Additional file 2).

## Discussion

The CRISPR/Cas9 system confers molecular immunity in bacterial and archaeal species, enabling these prokaryotes to fend off viral infections [42]. In this study, we demonstrated the portability of the CRISPR/Cas9 system to plants, where it is capable of conferring molecular immunity against invading DNA viruses. To evaluate the feasibility of using CRISPR/Cas9-mediated interference against DNA viruses in plants, we performed TRV-mediated systemic delivery of sgRNAs into NB-Cas9OE plants. Targeting of the coding and non-coding sequences of TYLCV resulted in specific modification of viral sequences, and subsequently attenuated viral replication and accumulation. Because binding of the CRISPR/Cas9 system to the IR can attenuate viral replication, we investigated the presence and efficiency of genomic modification at the IR. Our data demonstrate considerable levels of genomic modification at the IR, indicating that the Cas9 endonuclease was catalytically active. However, it remains to be determined whether binding of catalytically inactive Cas9 (dCas9) could interfere with virus replication and accumulation.

We tested sgRNAs targeting the CP, the RCRII motif of Rep, and the IR sequences, and compared their efficiency to interfere with TYLCV. All sgRNAs were capable of mediating targeted cleavage of the TYLCV genome. Extensive molecular analysis using RCA, semi-quantitative PCR, and Southern blotting assays revealed that targeting the IR led to a significant reduction in viral replication and accumulation relative to other TYLCV targets. Targeting of CP and RCRII attenuated symptoms to a lesser extent than targeting of the IR, possibly because both CP and RCRII encode proteins, and a small amount of protein may be sufficient for TYLCV to complete its cycle and develop symptoms. Furthermore, because the IR contains the stem-loop invariant sequence (TAATATTAC) of the TYLCV IR, occupation or modification of this region by Cas9 makes it inaccessible to Rep and/or other binding proteins responsible for viral replication.

Subsequently, we investigated whether the co-delivery of multiple sgRNAs via the TRV system would have an additive effect, leading to higher interference levels than obtained with single sgRNAs. The results demonstrated that targeting CP and IR using separate RNA2 genomes reduced viral replication and accumulation to levels similar to those obtained with a single sgRNA targeting either the IR or CP. Although we did not observe any additive effect on the accumulation of the TYLCV genome, this might be due to the nature of the TRV infection; i.e., because the two sgRNAs are not made in the same cell, they are unable to simultaneously cleave the TYLCV. On the other hand, construction of a TRV RNA2 genome containing multiple sgRNAs led to multiplexed editing in single cells, resulting in an additive effect.

One important criterion for in planta viral interference is specificity against particular viral strains. We investigated the specificity of our CRISPR/Cas9-mediated viral interference system using sgRNAs targeting either TYLCV or BCTV. When we used sgRNA targeting TYLCV sequences, only the TYLCV genome was modified, whereas when we used sgRNA targeting the BCTV, only the BCTV genome sequence was modified, demonstrating the specificity of interference. Such specificity is of particular importance when targeting newly evolved viral variants.

Engineered resistance of the plant host to viral infection might place selection pressure on the virus, leading to generation of variants with better adaptation, e.g., by altering the PAM tri-nucleotide sequence or abrogating recognition of the target sequence by the CRISPR/Cas9 system. For example, TYLCV recombinant variants that overcome resistance have emerged in Ty-I resistant plants [22]. Because the CRISPR/Cas9 system targets viral DNA for destruction, DNA repair can lead to the generation of viral variants with different PAM sequences that can evade the activity of the CRISPR/Cas9 system. Moreover, because particular DNA sequences (e.g., the IR) are key to viral replication, mutagenesis of these sequences will lead to significant reduction in viral replication [43]. To maintain the replication of a new viral variant, the target sequence must mutate to escape recognition by the CRISPR/Cas9 system, and the key replication enzymes that bind to this sequence must also mutate to recognize the new sequence. Targeting two viral sequences for cleavage will lead to the destruction of the viral genome, reducing the likelihood of DNA repair and generation of infectious viral variants. The emergence of variants can be combated through the design and application of sgRNA molecules specific for the new variant sequences.

We subsequently tested whether the CRISPR/Cas9 system is capable of targeting multiple viral strains by co-infiltration of sgRNAs targeting TYLCV, BCTV, and MeMV. The results showed that it was possible to target more than one virus with a single sgRNA. Recent work demonstrated the feasibility of expressing several sgRNAs simultaneously [41]. Such a system could be used to express sgRNAs with specificity for multiple DNA viruses, enabling engineering of plants resistant to multiple viruses or viral strains. This strategy should be effective in developing resistance to mixed infections. It should be noted that when using TRV-mediated delivery of sgRNAs targeting coding and noncoding sequences, the CRISPR/Cas9 system resulted in efficient interference of TYLCV. However, we observed less accumulation of the TYLCV genome when empty TRV vector was used in NB-Cas9OE plants challenged with TYLCV compared with NB-Cas9OE plants challenged with TYLCV but carrying no TRV vectors (Fig. 1d, e; Figure S12 in Additional file 2). Therefore, we do not entirely exclude other contributing silencing factors caused by the presence of TRV vector due to more complex phenomena at play, which requires further studies.

In summary, CRISPR/Cas9-mediated virus interference in plants has numerous important features, including: 1) the ability to target multiple DNA viruses simultaneously using a single sgRNA targeting a conserved sequence preceding the PAM tri-nucleotide sequence; 2) the capacity for multiplexed editing of single or multiple viruses using multiple sgRNAs; 3) the potential to overcome resistance by targeting newly evolved viral revertants with new sgRNAs; 4) applicability to all plant DNA viruses; and 5) applicability to all transformable plant species. Establishing the efficacy and extending the utility of the CRISPR/Cas9 system for viral interference in plants will create a platform for dissecting natural resistance and immune functions. At the same time, it will provide biotechnologists with a powerful tool for producing crop plants resistant to multiple viral infections.

## Conclusions

The data presented here show that the CRISPR/Cas9 system can be used for targeted interference and cleavage of the TYLCV genome. Targeting the TYLCV IR led to a significant reduction in TYLCV accumulation and disease symptoms. CRISPR/Cas9-mediated interference is virus strain-specific, and can therefore be used to target multiple viruses. Our results confirm the efficacy of the CRISPR/Cas9 system for virus interference, providing new possibilities for engineering plants resistant to DNA viruses.

## Materials and methods

### Vector construction

To clone sgRNAs targeting the TYLCV genome in the TRV RNA2 vector, we used a PCR-based restriction ligation procedure. A fragment containing the 20-nucleotide target sequence, the 84-bp Cas9 binding loop for sgRNA, and a 7 T repeat (as a terminator) was amplified by PCR. A forward primer containing an *Xba*I recognition site, 20-nucleotide target sequence, and 23-nucleotide Cas9-binding sgRNA scaffold was used with a reverse primer containing an *Xma*I recognition site to amplify a 116-bp PCR fragment. Primer sequences are provided in Table S1 in Additional file 1. The 116-bp PCR fragment of the sgRNA for each target was cloned into the TRV RNA2 vector under the control of the pea early browining virus promoter using the *Xba*I and *Xma*I restriction enzymes. Sanger sequencing was used to confirm all clone sequences.

### Mutation detection by restriction site loss

To test for targeted modification in the TYLCV genome by the CRISPR/Cas9 system, we subjected PCR products encompassing the target sequence to the restriction site loss assay. Genomic DNA was isolated from samples collected at 10 or 15 dpi. A fragment flanking the target region of TYLCV was PCR amplified using a specific primer set and Phusion high-fidelity polymerase (Supplementary sequence 1 in Additional file 4). DNA of PCR products was gel purified, and 200 ng was subjected to restriction enzyme protection analysis to detect the presence of indels. The digested products were separated on 2 % agarose gel. The PCR product was cloned into the pJet2.1 vector and subjected to Sanger sequencing.

### T7EI mutation detection assay

To determine and quantify the activity of the CRISPR/Cas9 system on the TYLCV genome, we measured mutations resulting from double strand break repair through the non-homologous end joining pathway, as described previously [36]. Briefly, genomic DNA was prepared from samples collected at 10 and 15 dpi and used as a template for PCR amplification of fragments encompassing the target sequences (Supplementary sequence 1 in Additional file 4). PCR amplicons were denatured, renatured, and treated with T7EI. To calculate the frequency of modification, the PCR amplicons were cloned into the pJET2.1 cloning vector and successful cloning was confirmed by colony PCR and *Bgl*II restriction digestion of the extracted plasmids. The percentage of modified clones was calculated after Sanger sequencing.

### RCA assay

Total genomic DNA extracted from plants was quantified using a NanoDrop spectrophotometer, adjusted to a concentration of 50 ng/μl, and analyzed using the RCA amplicon kit (GE Healthcare). Genomic DNA (50 ng) was incubated for 3 min at 95 °C in sample buffer and placed on ice for 5 min. Enzyme mix and reaction buffer were added, and the samples were incubated at 30 °C for 18 h for amplification, followed by incubation at 65 °C for 15 min to inactivate the enzyme. *Nco*I was added to the samples and incubated for 1 h at 37 °C, and the digested samples were resolved on 1 % agarose gels.

## Additional files

Additional file 1: Table S1. Primers used in this study. **Table S2** Summary of different sgRNA used for targeting of TYLCV genome. (PDF 3026 kb) Additional file 2: Figure S1. Dot blot analysis of the TYLCV genome accumulation in NBCas9OE. **Figure S2** CRISPR/Cas9-mediated virus interference in TYLCV sap inoculated plants. **Figure S3.** Targeting of CP region of TYLCV by CRISPR/Cas9. **Figure S4.** RCA analysis of the TYLCV genome accumulation. **Figure S5.** DNA blot analysis of the TYLCV genome accumulation. **Figure S6.** Reduction of TYLCV symptoms on NB-Cas9OE plants expressing IR-sgRNA. **Figure S7.** Reduction of TYLCV symptoms in NB-Cas9OE plants expressing CP-gRNA or RCRII-gRNA. **Figure S8.** Reduction of TYLCV symptoms in NB-Cas9OE plants coexpressing IR-sgRNA and CP-sgRNA. **Figure S9.** Restriction enzyme recognition site loss analysis from multiplexed targeting of IR and CP sequences. **Figure S10.** Alignment of the Sanger sequence reads of IR and CP regions of TYLCV from multiplexed targeting of IR and CP sequences. **Figure S11.** Recovery of TYLCV symptoms in NB-Cas9OE plants expressing IR-CP-gRNA. **Figure S12.** Southern blot analysis for the TYLCV genome accumulation. (PDF 3075 kb) Additional file 3:
**Supplementary methods.** (PDF 71 kb) Additional file 4:
**Supplementary sequences and maps.** **Supplemental sequence 1.** TYLCV 2.3 genome sequence and map. **Supplemental sequence 2.** IR-gRNA (TYLCV) sequence and map. **Supplemental sequence 3.** CP-gRNA (TYLCV) sequence and map. **Supplemental sequence 4.** RCRII-gRNA (TYLCV) sequence and map. **Supplementary sequence 5.** IR-gRNA (BCTV Worland) sequence and map. **Supplementary sequence 6.** RCRII-gRNA (BCTV Worland) sequence and map. **Supplementary sequence 7.** BCTV (Worland) sequence and map. **Supplementary sequence 8.** MeMV sequence and map. **Supplementary sequence 9.** Non-specific-sgRNA sequence and map. (PDF 3031 kb)

## Abbreviations

BCTV: beet curly top virus
bp: base pair
Cas: CRISPR-associated
CP: capsid protein
CR: common region
CRISPR: clustered regularly interspaced palindromic repeat
dpi: days post-infiltration
dsDNA: double-stranded DNA
IR: intergenic region
MeMV: Merremia mosaic virus
NB: *N. benthamiana*
OE: overexpressing
ORF: open reading frame
PAM: protospacer-associated motif
PCR: polymerase chain reaction
PTG: polycistronic tRNA–gRNA
RCA: rolling-circle amplification
sgRNA: single guide RNA
ssDNA: single-stranded DNA
TRV: tobacco rattle virus
TYLCV: tomato yellow leaf curl virus
