# Supplementary material for: CRISPR/Cas9-mediated viral interference in plants
Source: Genome Biol. 2015 Nov 11;16:238. doi: 10.1186/s13059-015-0799-6 (PMC4641396; doi:10.1186/s13059-015-0799-6)
Supplement: Additional file 1: Table S1. — Primers used in this study. Table S2 Summary of different sgRNA used for targeting of TYLCV genome. (PDF 3026 kb) [file 13059_2015_799_MOESM1_ESM.pdf]

SUPPLEMENTARY INFORMATION: ADDITIONAL FILE 1

CRISPR/Cas9-mediated viral interference in plants

Zahir Ali[1], Aala Abulfaraj[1], Ali Idris[1], Shakila Ali[1], Manal Tashkandi[1], and Magdy M. Mahfouz[1, 2]

[1] Laboratory for Genome Engineering, Center for Desert Agriculture & Division of Biological Sciences, 4700 King Abdullah University of Science and Technology, Thuwal 23955-6900, Saudi Arabia

Supplementary Tables

Supplementary Table 1. Primers used in this study.

Supplementary Table 2. Summary of different sgRNA used for targeting of TYLCV genome.

Supplementary Table 1. Primers used in this study**.**

primers name	sequence (5ˉ ---- 3ˉ)	Usage
TYLCV2.3-IR-T-F	AATTGGGAAAGTGCTTCCTCT	Simi-q PCR, to amplify TYLCV IR flanking region, to make probe for southern, detection TYLCV by PCR
TYLCV2.3-IR-T-R	ATAGTCACGGGCCCTTACAACA	
TYLCV-IR-T1	CGAGTCTAGAGGCCATCCGTATA ATATTACGTTTTAGAGCTAGAAA TAGCAAG	To clone TYLCV IR-gRNA
SPDK-gRNA-R	acatGCCCGGgAAAAAAGCACCG ACTCGG	To clone all gRNA
NB-ACTIN1-RT-F	TGAAGATCCTCACAGAGCGTGG	RT-PCR normalization control
NB-ACTIN1QRT-LIU-R	TTGTATGTGGTCTCGTGGATTC	
TYLCV-CP-T1	CGAGTCTAGAGCTTCGGCGAACC TTCGAGACGTTTTAGAGCTAGAA ATAGCAAG	To clone TYLCV CP-gRNA
TYLCV-RCRII-T	CGAGTCTAGAGTGGATGAGCACA TGCAAGTGGTTTTAGAGCTAGAA ATAGCAAG	To clone TYLCV RCRII-gRNA
TRV1-RELICASE-RT-F	CTACTGGGAGAGCAGCAACC	For detection of TRV-RNA1 systemic movement
TRV1-REPLICASE-RT-R	CTGAGCGCAAAAGTACACCA	
TRV2-CP-RT-F	TTGGGTGGAATCAGTTTCGT	For detection of TRV-RNA2 systemic movement
TRV2-CP-RT-R	TCTTCCAAAGTCGAGCCAGT	
WOR-IR-T-F	GGCTTTAATTTGAAATGATGGTG	For PCR flanking Worland IR target
WOR-IR-T-R	AAAAATTCGTACCTGATTGCAG	
WOR-IR-T1	CGAGTCTAGAGCCATCCGCAATAATATTACG TTTTAGAGCTAGAAATAGCAAG	To clone Worland IR-gRNA
WOR-L1-T1/2-F	TGCTTCAGCTGCATTACCTG	For PCR flanking Worland RCRII target
WOR-L1-T1/2-R	ATGGCCCCTGGAGGTATATAAG	
WOR-LI-RCRII-T	GAGTCTAGAGCTTTGAATTGGATGAGGGCG GTTTTAGAGCTAGAAATAGCAAG	To clone Worland RCRII-gRNA
TYLCV 2.3-CP-T1/2-F	TTCTTCACGGTTGCGGTACT	For PCR flanking TYLCV CP target
TYLCV2.3-CP-T1/2-R	GAGCTTTGGACCCTGAATTG	
TYLCV2.3-REP-T1/2-F	GAGCTTTGGACCCTGAATTG	For PCR flanking TYLCV RCRIII target
TYLCV2.3-REP-T1/2-R	TTGGAGCGTGATGATTTTGA	
MeMV-IR-T-F	TGTGCAGAGCTTTGATTTGG	For PCR flanking MeMV IR target
MeMV-IR-T-R	AAATTGGCGTTGCGACTAAC	

Treatment / sgRNA	Mock experiment	Vector Control experiment	IR-sgRNA / TYLCV	CP-sgRNA / TYLCV	RCRII-sgRNA / TYLCV	IR + CP-sgRNA / TYLCV	IR-CP-sgRNA / TYLCV (PTG)
**No. of plants / experiment**	8	8	12	8	8	8	10
**No. of experimental Repeats**	3	3	5	3	3	3	4
**Total number of plants**	40	40	60	24	24	24	40
**No. of Plants with no TYLCV symptoms on leaves**	(100 %)	(None)	(85 %)	(None)	(11.6 %)	(88.6 %)	(95 %)
**No. of Plants with no TYLCV symptoms on leaves but stunted**	(None)	(None)	(35.8 %)	(None)	(None)	(27.7 %)	(7 %)
**No. of Plants with Mild TYLCV symptoms**	(None)	(None)	(15.2 %)	(73.3 %)	(88.7 %)	(11.3 %)	3 %
**No. of Plants with severe TYLCV symptoms**	(None)	(100 %)	(None)	(27.7 %)	(12.3 %)	(None)	(None)
**TYLCV detection by PCR**	(None)	(100 %)	(39 %)	(94 %)	(91 %)	(25 %)	(11 %)
**TYLCV detection by DNA blotting**	(None)	(100 %)	(19 %)	(68 %)	(53 %)	(11 %)	(3 %)
**No. of clones sequenced**	(None)	30	300	88	142	98(IR) / 58 (CP)	112(IR) / 66(CP)
**No. of clones with Indels**	(None)	(None)	(36 - 42 %)	(22 – 28 %)	(31 -39 %)	28 % (IR) and 19 % (CP)	38 % IR and 31 % CP
**Can target**	N.A	N.A	IR sequence of TYLCV (38%) BCTV (22%) MeMV (31%)	Specific	Specific	N.A	N.A

Supplementary Table 2. Summary of different sgRNA used for targeting of TYLCV genome. All the observations were performed with NB-Cas9OE plants. Data were collected at 28 dpi. Three biologically independent experiments were performed with at least 8 replicate plants for each type of sgRNA. Mock, NB-Cas9OE plants treated with infiltration buffer; Vector Control, TRV2 with a non-viral target sequence;N.A not applicable.
