# Supplementary material for: CRISPR/Cas9-mediated viral interference in plants
Source: Genome Biol. 2015 Nov 11;16:238. doi: 10.1186/s13059-015-0799-6 (PMC4641396; doi:10.1186/s13059-015-0799-6)
Supplement: Additional file 2: Figure S1. — Dot blot analysis of the TYLCV genome accumulation in NBCas9OE. Figure S2 CRISPR/Cas9-mediated virus interference in TYLCV sap inoculated plants. Figure S3. Targeting of CP region of TYLCV by CRISPR/Cas9. Figure S4. RCA analysis of the TYLCV genome accumulation. Figure S5. DNA blot analysis of the TYLCV genome accumulation. Figure S6. Reduction of TYLCV symptoms on NB-Cas9OE plants expressing IR-sgRNA. Figure S7. Reduction of TYLCV symptoms in NB-Cas9OE plants expressing CP-gRNA or RCRII-gRNA. Figure S8. Reduction of TYLCV symptoms in NB-Cas9OE plants coexpressing IR-sgRNA and CP-sgRNA. Figure S9. Restriction enzyme recognition site loss analysis from multiplexed targeting of IR and CP sequences. Figure S10. Alignment of the Sanger sequence reads of IR and CP regions of TYLCV from multiplexed targeting of IR and CP sequences. Figure S11. Recovery of TYLCV symptoms in NB-Cas9OE plants expressing IR-CP-gRNA. Figure S12. Southern blot analysis for the TYLCV genome accumulation. (PDF 3075 kb) [file 13059_2015_799_MOESM2_ESM.pdf]

# SUPPLEMENTARY INFORMATION: ADDITIONAL FILE 2

## CRISPR/Cas9-mediated viral interference in plants

Zahir Ali[1], Aala Abulfaraj[1], Ali Idris[1], Shakila Ali[1], Manal Tashkandi[1], and Magdy M. Mahfouz[1, 2]

[1] Laboratory for Genome Engineering, Center for Desert Agriculture & Division of Biological Sciences, 4700 King Abdullah University of Science and Technology, Thuwal 23955-6900, Saudi Arabia

**Supplementary Figures and Legends**

**Supplementary Figure 1**. Dot blot analysis of the TYLCV genome accumulation in NB-Cas9OE.

**Supplementary Figure 2.** CRISPR/Cas9-mediated virus interference in TYLCV sap inoculated plants.

**Supplementary Figure 3.** Targeting of CP region of TYLCV by CRISPR/Cas9.

**Supplementary Figure 4.** RCA analysis of the TYLCV genome accumulation.

**Supplementary Figure 5.** DNA blot analysis of the TYLCV genome accumulation.

**Supplementary Figure 6.** Reduction of TYLCV symptoms on NB-Cas9OE plants expressing IR-sgRNA.

**Supplementary Figure 7.** Reduction of TYLCV symptoms on NB-Cas9OE plants expressing CP-gRNA or RCRII-gRNA.

**Supplementary Figure 8.** Reduction of TYLCV symptoms on NB-Cas9OE plants co-expressing IR-sgRNA and CP-sgRNA.

**Supplementary Figure 9.** Restriction enzyme recognition site loss analysis from multiplexed targeting of IR and CP sequences.

**Supplementary Figure 10.** Alignment of the Sanger sequence reads of IR and CP regions of TYLCV from multiplexed targeting of IR and CP sequences.

**Supplementary Figure 11.** Recovery of TYLCV symptoms in NB-Cas9OE plants expressing IR-CP-gRNA.

**Supplementary Figure 12.** Southern blot analysis for the TYLCV genome accumulation.

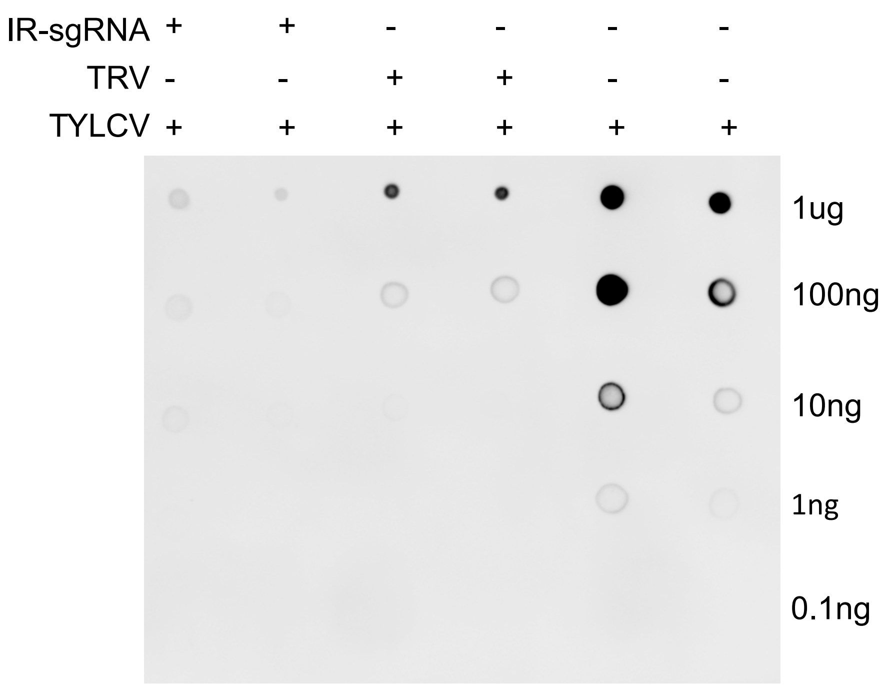


Supplementary Figure 1. Dot blot analysis of the TYLCV genome accumulation.

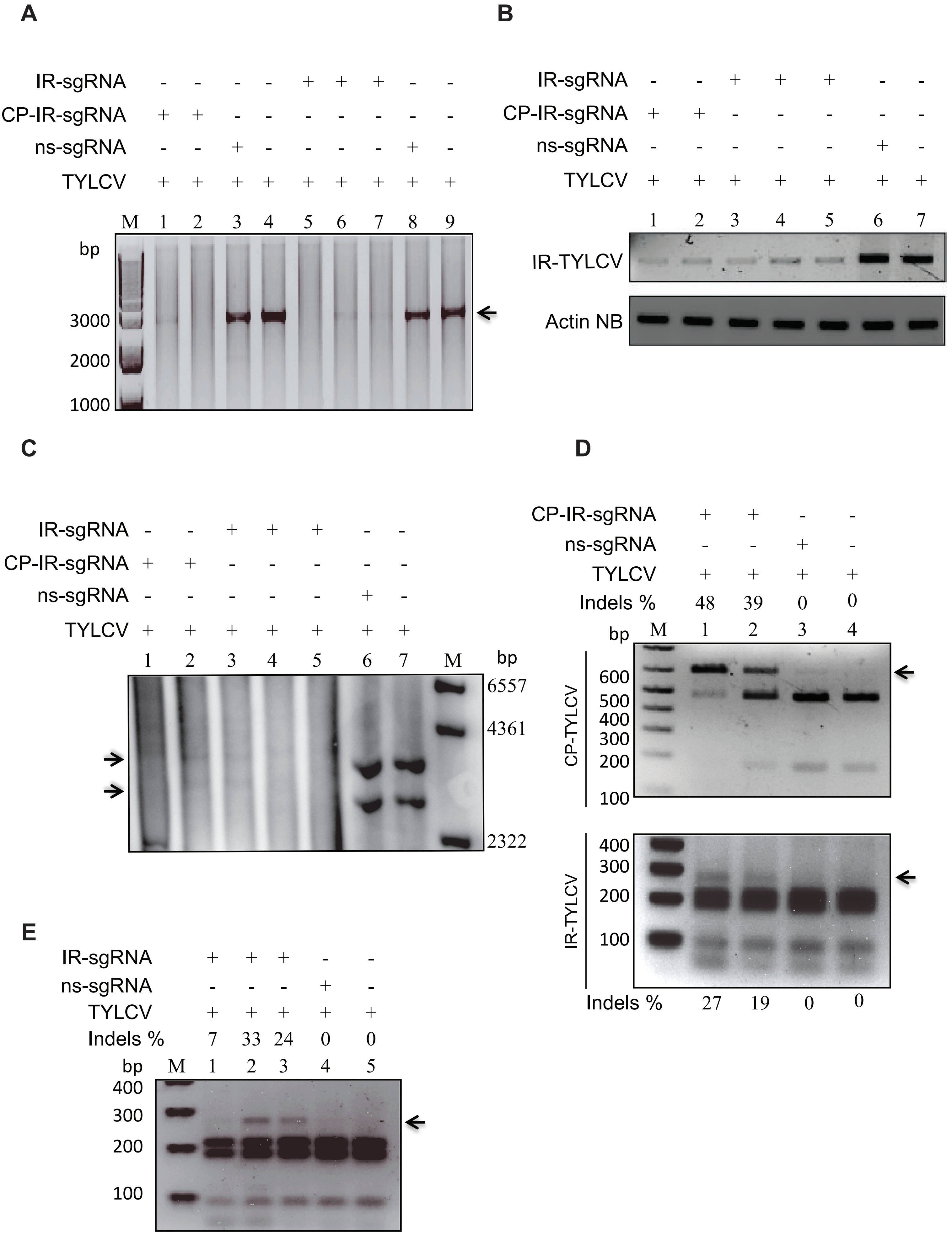


Supplementary Figure 2. CRISPR/Cas9-mediated virus interference in TYLCV sap inoculated plants.

**A**

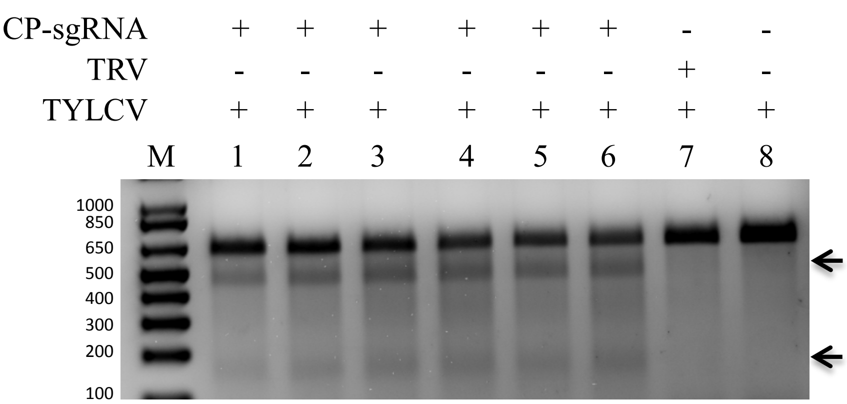


**B**

```
                                                   PAM
                                             BsmBI
WT    GGCTGTCGAAGTTCAGCCTTCGGCGAACCTTCGAGACGGGCGTGGAAATGATTATATCGC
C06   GGCTGTCGAAGTTCAGCCTTCG--------TCGAGACGGGCGTGGAAATGATTATATCGC -08
C09   GGCTGTCGAAGTTCAGCCTTC-----------GAGACGGGCGTGGAAATGATTATATCGC -11
F01   GGCTGTCGAAGTTCAGCCTTCGGCGAAGCTTCGAGACGGGCGTGGAAATGATTATATCGC C>G
```

Supplementary Figure 3. Targeting of TYLCV CP sequence by CRISPR/Cas9

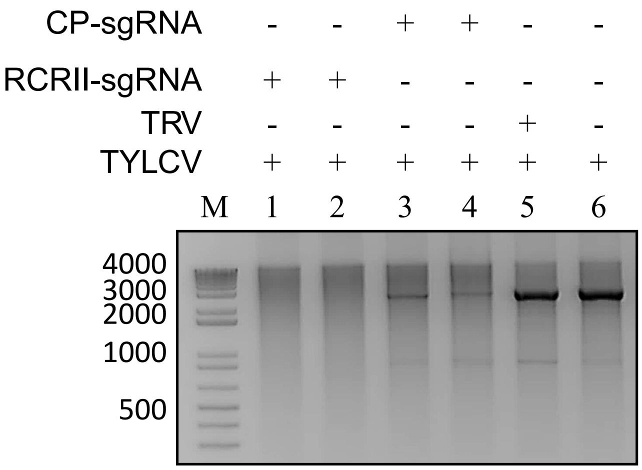


Supplementary Figure 4. RCA analysis of the TYLCV genome accumulation.

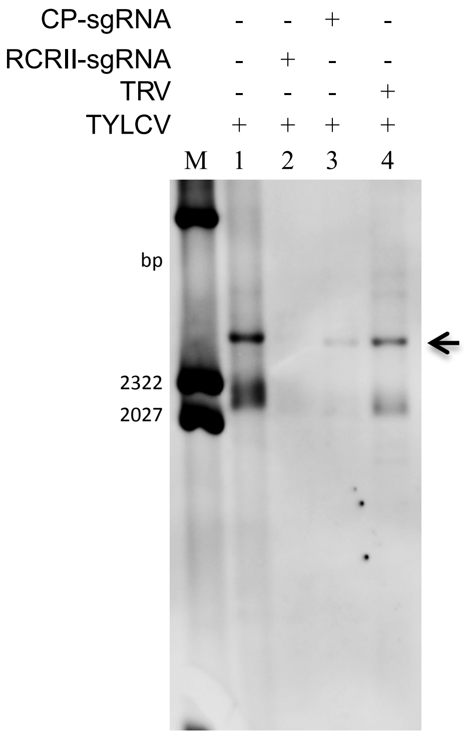


Supplementary Figure 5. DNA blot analysis of the TYLCV genome accumulation.

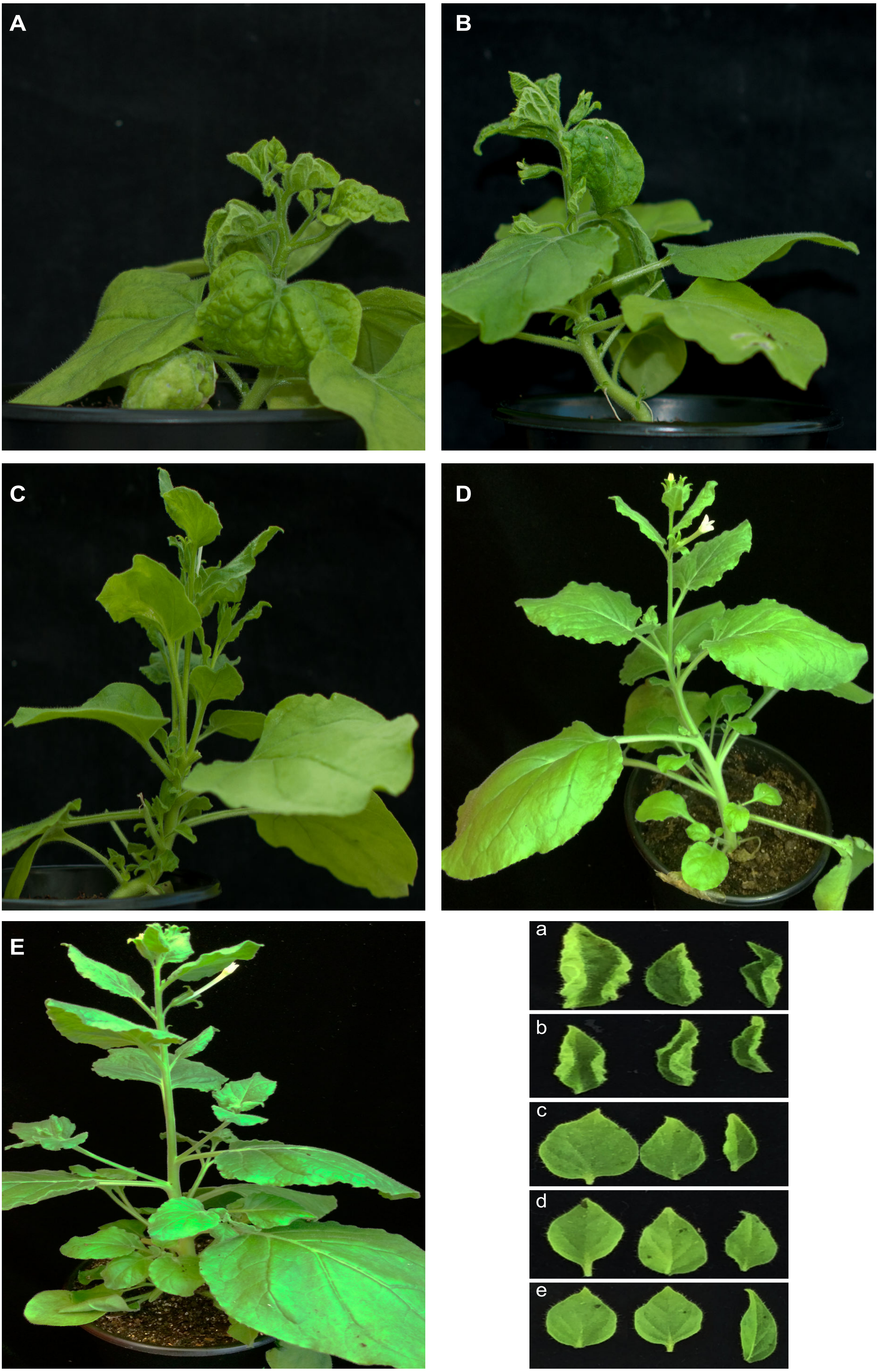


Supplementary Figure 6. Reduction of TYLCV symptoms  on NB-Cas9OE plants expressing IR-sgRNA.

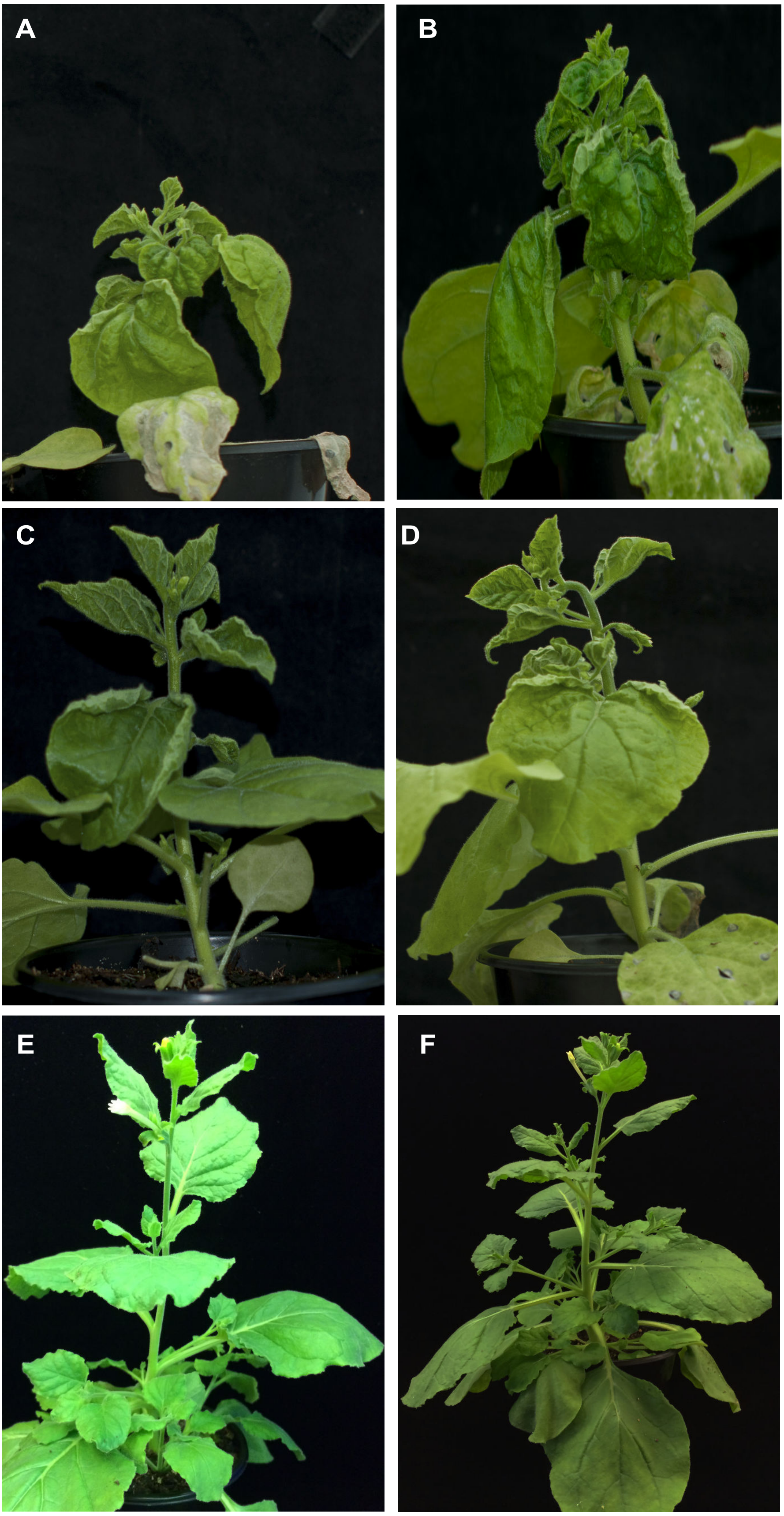

Supplementary Figure 7. Reduction of TYLCV symptoms on NB-Cas9OE plants expressing CP-gRNA or RCRII-gRNA.

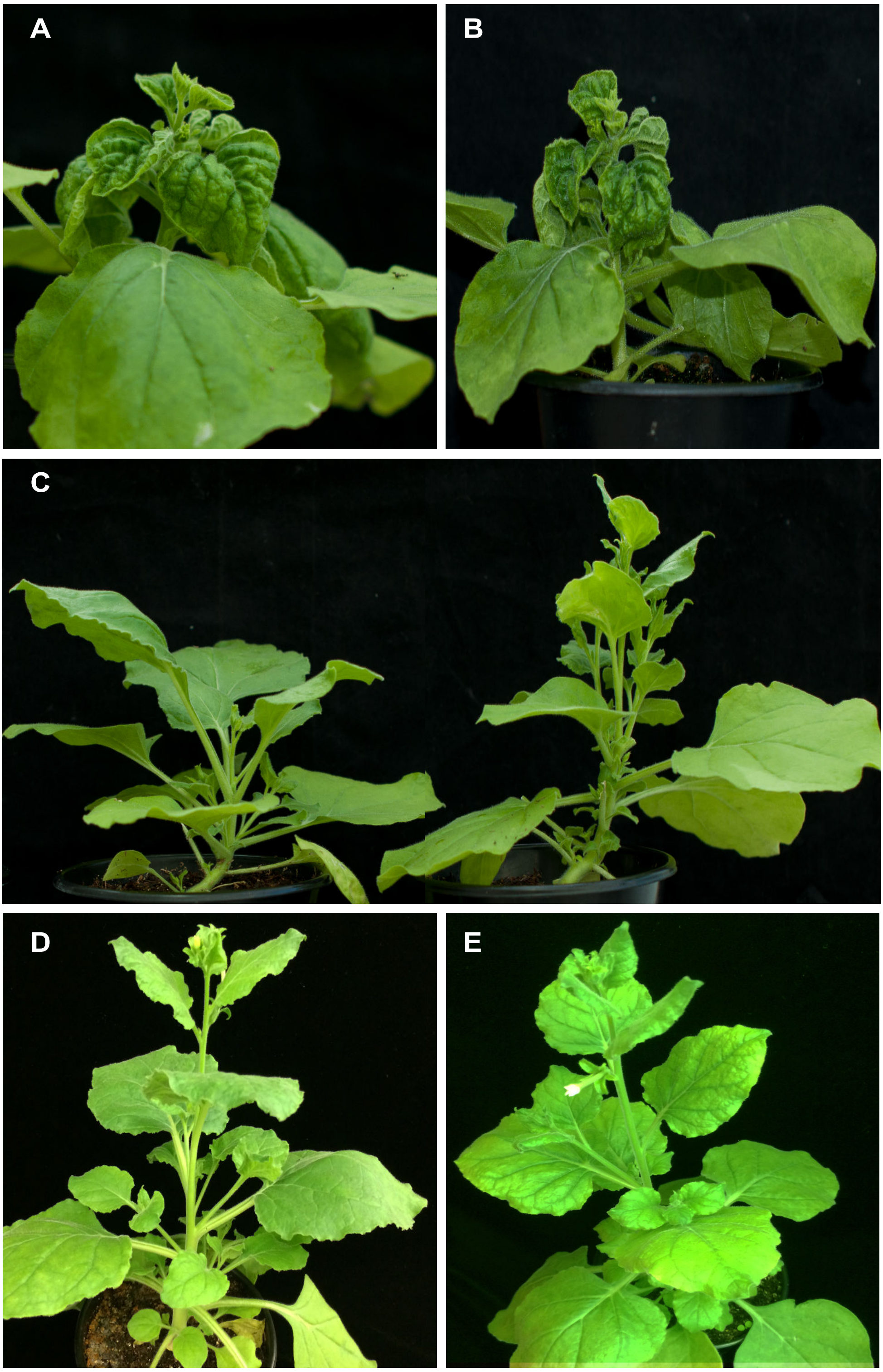


Supplementary Figure 8. Reduction of TYLCV symptoms NB-Cas9OE plants co-expressing IR-sgRNA and CP-sgRNA.

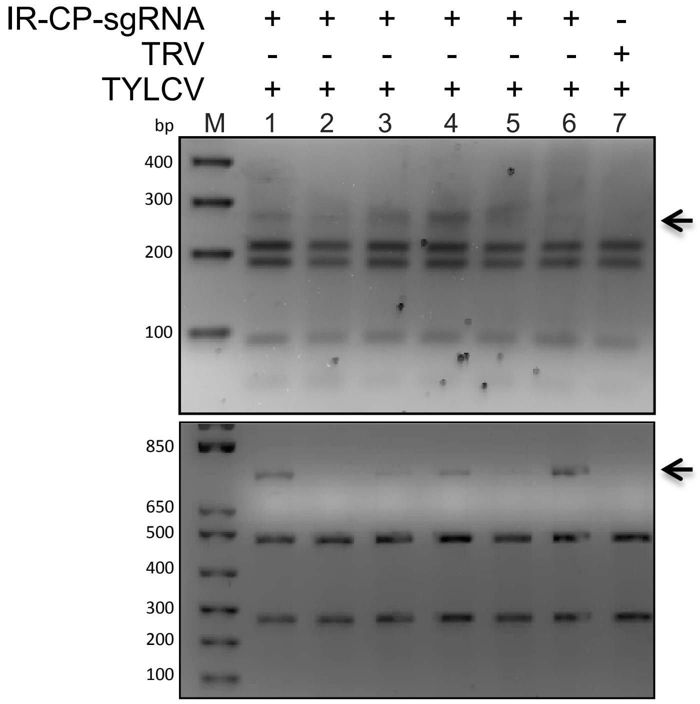


Supplementary Figure 9. Restriction enzyme recognition site loss analysis from multiplex targetting of IR and CP sequences.

**A**

```
                                        SspI     PAM
WT     TCCAAAAAGCGGCCATCCGTATAATATTACCGGATGGCCGCGA
B02    TCCAAAA--------------------TACCGGATGGCCGCGA    -20
B03    TCCAAAAAGCGGCCATCCGTATAATAT--------------GA    -14
C07    TCCAAAAAGCGGCCATCCGTATAATA----------------A    -16
H08    TCCAAAAAGCGG---------------TACCGGATGGCCGCGA    -15
G02    TCCAAAAAGCGGCCATCCGTATAATATATTACCGGATGGCCGAAT  +2
```

**B**

```
                                        BsmBI  PAM
WT     GAAGTTCAGCCTTCGGCGAACCTTCGAGACGGGCGTGGAAATG
E07    GAAGTTCAGCCTT----------------CGGCCGTGGAAATG   -16
C04    GAAGTTCAGCCTTCGG-------------CGGGCGTGGAAATG   -13
A06    GAAGTTCAGCCTTCGGCGA------------GGCGTGGAAATG   -12
B06    GAAGTTCAGCCTTCGGCGAAC-----AGACGGGCGTGGAAATG   -5
A07    GAAGTTCAGCCTTCGGCGAACCTTCGAAGACGGGCGTGGAAATG  +1
```

Supplementary Figure 10. Alignment of the Sanger sequence reads of IR and CP regions of TYLCV from multiplexed targetting of IR and CP sequences.

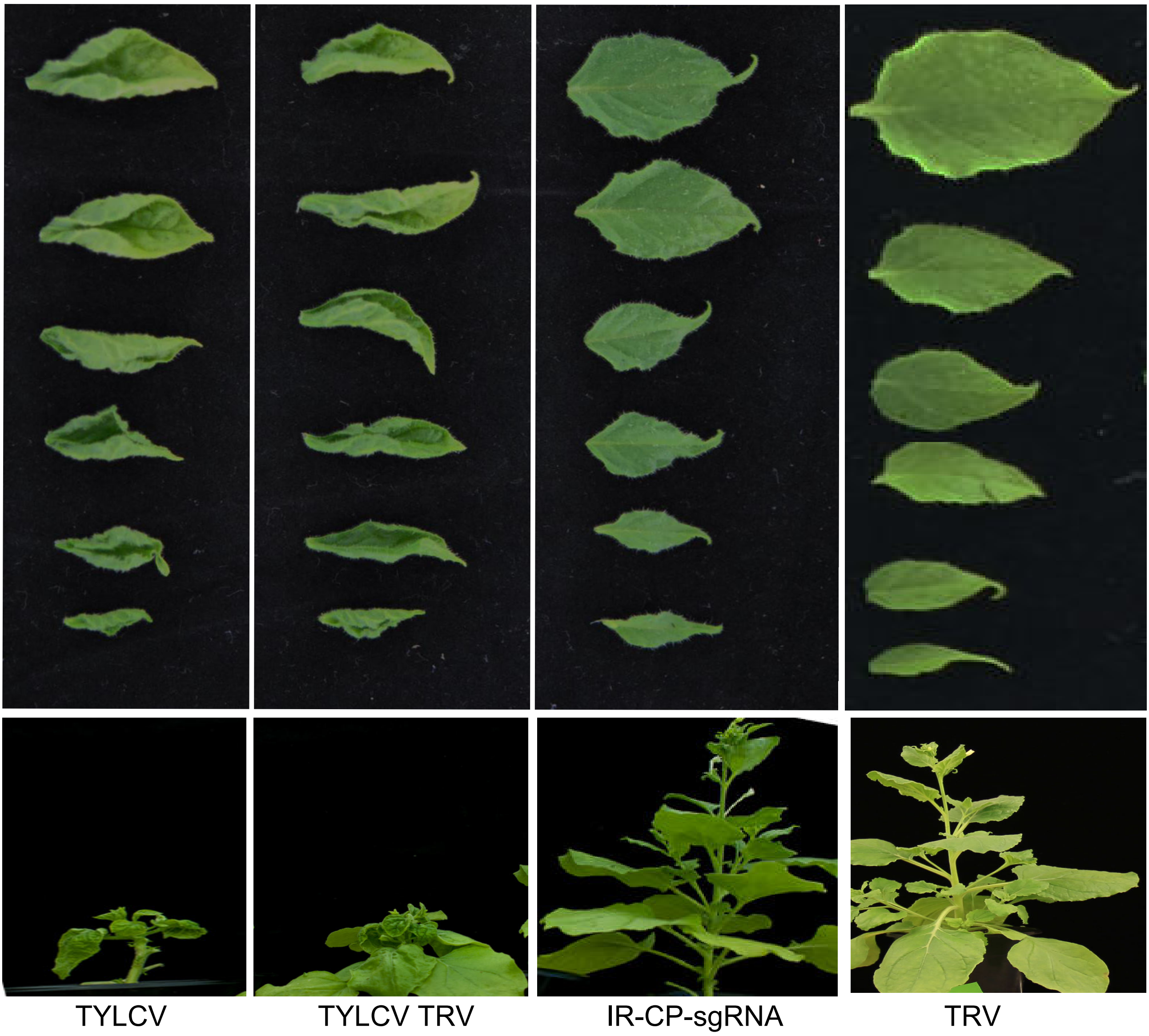


Supplementary Figure 11. Recovery of TYLCV symptoms of NB-Cas9OE plants expressing IR-CP-sgRNA.

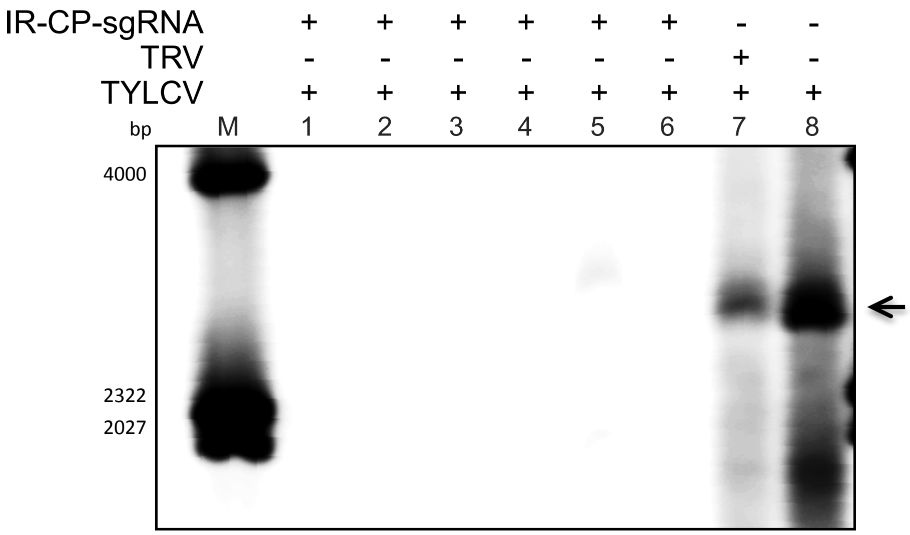


Supplementary Figure 12. DNA blot analysis for the TYLCV genome accumulation from multiplexed targetting of IR and CP sequences.

**Supplementary Figures legends,**

**Supplementary Figure 1. Dot blot analysis of the TYLCV genome accumulation in NB-Cas9OE.** Total genomic DNA (diluted to 1 µg, 100ng, 10ng, 1ng and 0.1ng) isolated from NB-Cas9OE plants inoculated with TRV containing IR-sgRNA and subsequently infected with TYLCV was blotted to nylon membrane and probed with DIG-labeled probe against the 560 bp of the IR region. Detection of the probe with anti-DIG antibody demonstrate lower accumulation of the of TYLCV genome in plants expressing IR-sgRNA compared to controls. TRV RNA2 empty vector with TYLCV and TYLCV alone were used as controls.

**Supplementary Figure 2. CRISPR/Cas9-mediated virus interference in TYLCV sap inoculated plants.**

(A) Assay for rolling-circle amplification (RCA) of TYLCV genome in sap inoculated plants. TYLCV genomic DNA accumulation in sap inoculated plants with established IR-sgRNA or CP-IR-sgRNA was reduced relative to plants inoculated with non-specific sgRNA (ns-sgRNA) or TYLCV alone. (B) Semi-quantitative PCR of TYLCV genomic DNA. TYLCV inoculation into Cas9OE plants with established IR-sgRNA or CP-IR-sgRNA accumulated lower levels of TYLCV than plants inoculated with ns-sgRNA or TYLCV alone. Genomic DNA from *N. benthamiana* actin was used as a normalization control. (C) DNA blot analysis assaying for the accumulation of TYLCV genomic DNA in Cas9OE plants. TYLCV genomic DNA was detected with a DIG-labeled probe against 560 bp encompassing the IR region. Individual plants expressing IR-sgRNA or IR-CP-sgRNA that were sap inoculated with TYLCV exhibited reduced accumulation of the TYLCV genome relative to plants inoculated with ns-sgRNA and TYLCV alone. (D) Mutation analysis by restriction site loss assay in plants expressing PTG for CP and IR and challenged with TYLCV via sap inoculation. The TYLCV CP and IR resistant fragments were detected for the loss of the BsmBI and *Ssp*I recognition sequences at the targeted locus, respectively, but not in samples from ns-sgRNA and TYLCV alone control plants. (E) Mutation analysis by restriction site loss assay of plants expressing IR-sgRNA and challenged with TYLCV via sap inoculation. The loss of the *Ssp*I site was detected at the targeted locus but not in samples from control plants. Arrows in A, C, D and E indicate the respective DNA fragments resistant to enzymatic digestion.

**Supplementary Figure 3. Targeting the TYLCV CP sequence using the CRISPR/Cas9 system.** (A) T7EI assay for detection of indels at CP sequence of the TYLCV genome. T7EI

digested only PCR-amplicons from plants infiltrated with CP-sgRNA specific to CP sequence, indicating the presence of targeted modification, but could not digest the CP sequence in the controls. (B) Alignment of Sanger-sequencing reads of amplicons encompassing the TYLCV-CP region. Wild-type (WT) TYLCV sequences are shown at the top (target sequence is shown in red, the protospacer-associated motif [PAM] is indicated by green dots, followed by the various indels formed, indicated by numbers to the right of the sequence [-, deletion of x nucleotides; +, insertion of x nucleotides; and >, change of x nucleotides to y nucleotides]

**Supplementary Figure 4. RCA analysis of the TYLCV genome accumulation.** NB-Cas9OE plants expressing either CP-sgRNA or RCRII-sgRNA when infiltrated with TYLCV, accumulated a reduced amount of TYLCV genome compared to empty TRV control plants.

**Supplementary Figure 5. DNA blot analysis of the TYLCV genome accumulation.** Total genomic DNA extracted from NB-Cas9OE plants expressing either CP-sgRNA or RCRII-sgRNA was used to assess the TYLCV genome accumulation with a DIG-labeled probe against the 560 bp IR region. TYLCV accumulation was reduced in both CP-sgRNA and RCRII-sgRNA expressing plants compared to TYLCV with empty TRV vector or TYLCV alone controls

**Supplementary Figure 6. Reduction of TYLCV symptoms on NB-Cas9OE plants expressing the IR-sgRNA.** Compared to plants challenged with TYLCV alone (A, a) or TYLCV with empty TRV (B, b), NB-Cas9OE plants expressing IR-sgRNA (C, c) exhibited reduced symptoms. (D, d) Cas9OE plants infiltrated with TRV only. (E, e) Mock infiltrated Cas9OE plants. Representative three leaves from the top of each plant are shown with small letters a, b, c, d and e. Photos are representative of a single set of three experimental repeats with eight plants at least for each treatment. Photos were taken at 28 days post-infiltration. For more details see Supplementary table 2.

**Supplementary Figure 7. Reduction of TYLCV symptoms in NB-Cas9OE plants expressing either CP-gRNA or RCRII-gRNA.** Compared to plants treated with TYLCV alone (A) or TYLCV with empty TRV (B), NB-Cas9OE plants expressing CP-sgRNA (C) and RCRII-sgRNA (D) exhibited lower TYLCV symptoms. Leaves are less curly and plants are growing (E)

Cas9OE plants infiltrated with TRV only. (F) Mock infiltrated Cas9OE plants. Photos were taken at 28 days post-infiltration. Photos are representative of a single set of three experimental repeats with eight plants at least for each treatment.

**Supplementary Figure 8. Reduction of TYLCV symptoms on NB-Cas9OE plants co-expressing IR-sgRNA and CP-sgRNA.** Cas9OE plants were co-infiltrated with agrobacterium containing TRV RNA1 and RNA2 with sgRNA against IR or CP respectively. Co-infiltration of IR and CP sgRNAs exhibited similar recovery of symptoms as single IR-sgRNA (Supplementary Figure 5). But compared to plants challenged with TYLCV alone (A) or TYLCV with empty TRV (B), NB-Cas9OE plants expressing IR-sgRNA and CP-sgRNA exhibited significantly reduced TYLCV symptoms on leaves (C). (D) Cas9OE plants infiltrated with TRV only. (F) Mock infiltrated Cas9OE plants. Photos were taken at 28 days post-infiltration. Photos are representative of a single set of three experimental repeats with eight plants at least for each treatment.

**Supplementary Figure 9. Restriction enzyme recognition site loss analysis from multiplexed targeting of IR and CP sequences.** Cas9OE plants were infiltrated with agrobacterium containing TRV RNA1 and RNA2 with a tRNA processing cassette (PTG) expressing both IR and CP sgRNA. DNA was extracted from the systemic leaves and PCR amplicons of IR region were digested with SspI, and CP region amplicons were digested with BsmbI. The arrow indicated the SspI resistant DNA fragments.

**Supplementary Figure 10. Alignment of the Sanger sequence reads of amplicons encompassing the TYLCV IR and CP sequences from multiplexed targeting.** Multiplex targeting of the TYLCV genome using TRV RNA2 genome engineered to simultaneously express the IR and CP gRNAs. PCR amplicons of the IR and CP region were cloned in pJET2.1 cloning vector and subjected to Sanger sequencing. Alignment of Sanger-sequencing reads of TYLCV IR and CP amplicons simultaneously targeted by IR- and CP- gRNAs.

**Supplementary Figure 11. Recovery of TYLCV symptoms in NB-Cas9OE plants expressing IR-CP-gRNA.** Compared to plants treated with TYLCV alone or TYLCV with

empty TRV, leaves of plants infiltrated with a PTG expressing both IR-gRNA and CP-gRNA exhibited significantly reduced TYLCV symptoms, and recovered normal growth pattern like TRV infiltrated plants. Photos were taken at 28 days post-infiltration. Photos are representative of a single set of three experimental repeats with eight plants at least for each treatment.

**Supplementary Figure 12. Southern blot analysis for the TYLCV genome accumulation.** Total genome DNA extracted from NB-Cas9OE plants expressing IR-CP-gRNA was detected for TYLCV genome accumulation with a DIG-labeled probe against the 560 bp IR region. TYLCV accumulation was not detected in IR-CP-gRNA expressing plants compared to TYLCV with empty vector or TYLCV alone. Arrow head indicate the TYLCV expected genome size in the TYLCV and TYLCV and TRV infiltrated plants.
