# Supplementary material for: CRISPR/Cas9-mediated viral interference in plants
Source: Genome Biol. 2015 Nov 11;16:238. doi: 10.1186/s13059-015-0799-6 (PMC4641396; doi:10.1186/s13059-015-0799-6)
Supplement: Additional file 3: — Supplementary methods. (PDF 71 kb) [file 13059_2015_799_MOESM3_ESM.pdf]

SUPPLEMENTARY INFORMATION: ADDITIONAL FILE 3

CRISPR/Cas9-mediated viral interference in plants

Zahir Ali[1], Aala Abulfaraj[1], Ali Idris[1], Shakila Ali[1], Manal Tashkandi[1], and Magdy M. Mahfouz[1, 2]

[1] Laboratory for Genome Engineering, Center for Desert Agriculture & Division of Biological Sciences, 4700 King Abdullah University of Science and Technology, Thuwal 23955-6900, Saudi Arabia

Additional methods

Agroinfiltration. Binary constructs, including TRV RNA2 harboring the gRNA, TRV RNA1, and TYLCV infectious clone 2.3, were individually transformed into *Agrobacterium tumefaciens* strain GV3103 by electroporation. Transformed single colonies were grown overnight in selective medium, re-suspended, and diluted to OD_{600} ~0.3 into infiltration medium (10 mM MES [pH 5.7], 10 mM $CaCl_2$, and 200 µM acetosyringone), and cultures were incubated at ambient temperature in the dark for 2–4 h. Subsequently, bacterial cultures were mixed prior to infiltration at a ratio of 1:1 (when two bacterial cultures were used) or 1:1:1 (when three bacterial cultures were used). Mixed bacterial cultures were infiltrated into the lower side of 3–4-week-old leaves of *N. benthamiana* Cas9-OE plants using a needleless 2 mL syringe. Leaf disc samples were collected from inoculated and systemic leaves 5, 10, 15, and 30 dpi and subjected to various molecular analyses to determine viral load and targeted modification of the viral sequence.

Semi-quantitative RT-PCR. Genomic DNA was extracted from plant samples as previously described (36). DNA was quantitated using a NanoDrop spectrophotometer and adjusted to 50 ng/µL. A 560 bp fragment was amplified from the TYLCV genome by PCR using a specific primer set (TYLCV2.3-IR-T-F and TYLCV2.3-IR-T-R) and 23–25 cycles with Phusion polymerase (New England Biolabs). A primer set (NB.ActinF and NB.ActinR) that amplifies the genomic DNA corresponding to *N. benthamiana* actin was used as a normalization control.

DNA blot analysis. A 560 bp DIG-labeled probe specific for the TYLCV sequence was prepared by PCR amplification of the region flanking the IR using a primer set (TYLCV2.3-IR-T-F and TYLCV2.3-IR-T-R)). Total genomic DNA (2 µg) from *N. benthamiana* plants was electrophoresed on 1% gels alongside the DIG-labeled size marker DIG marker-II (Roche). The DNA bands were transferred to a nylon membrane (Roche). Subsequently, the membrane was hybridized with the DIG-labeled probe, and DNA bands corresponding to TYLCV were visualized using alkaline phosphatase-conjugated anti-DIG (1:10,000) and CPD chemiluminescent substrate using an Alpha Innotech digital imaging system.

TRV-mediated delivery of sgRNA.

TRV VIGS vectors were previously used with TYLCV to discover host plant genes involved in the infection of TYLCV (Czosnek et al. 2013). Very recently, we have developed TRV as

an efficient vector for the systemic expression and delivery of sgRNA for the CRISPR/Cas9 system to mediate plant genome modification (Ali et al. 2015). Here, we employed the TRV system to deliver sgRNAs in Cas9OE in the TYLCV interference experiments. Briefly, three weeks old Cas9OE *N benthamiana* plants were co-infiltrated with agrobacterium cultures containing TRV RNA1 and RNA2 engineered to express sgRNAs against coding or non-coding sequence of TYLCV. Seven days post TRV-mediated sgRNA delivery, a second leaf was infiltrated with agrobacterium containing an infectious clone of TYLCV. Plants were placed in greenhouse for 10 days to develop TYLCV infection. Ten days post-TYLCV challenge, systemic leaves were collected for different molecular analyses. Photos were taken 28 days post TYLCV challenge to assess for TYLCV symptoms development or interference.

TYLCV inoculation via sap transmission.

TYLCV- sap inoculation was conducted as previously described by Gilbertson et. al. (1991). Briefly, two weeks old wild type *N.benthamiana* plants were infiltrated with *Agrobacterium tumefaciens* containing an infectious clone of TYLCV2.3. Systemic young infected leaves were grinded to fine powder in liquid nitrogen. Grinded leaves powder was re-suspended in 1: 4 w/v potassium phosphate buffer (0.1 M, pH 8.0). Next, the adaxial surface of three leaves and their pedicels of *N. benthamiana* Cas9OE plants with established TRV sgRNAs including IR-sgRNA, CP-IR-sgRNA, and non-specific sgRNA (ns-sgRNA) were dusted with Carborundum (200- 450 mesh). Sap was applied to the entire adaxial surface dusted with Carborundum by thoroughly rubbing sap-dipped pestle in circular motion. Systemic leaves were collected after 21 of sap application and DNA was extracted for molecular analysis for virus interference.
