# Supplementary material for: CRISPR/Cas9-mediated viral interference in plants
Source: Genome Biol. 2015 Nov 11;16:238. doi: 10.1186/s13059-015-0799-6 (PMC4641396; doi:10.1186/s13059-015-0799-6)
Supplement: Additional file 4: — Supplementary sequences and maps. Supplemental sequence 1. TYLCV 2.3 genome sequence and map. Supplemental sequence 2. IR-gRNA (TYLCV) sequence and map. Supplemental sequence 3. CP-gRNA (TYLCV) sequence and map. Supplemental sequence 4. RCRII-gRNA (TYLCV) sequence and map. Supplementary sequence 5. IR-gRNA (BCTV Worland) sequence and map. Supplementary sequence 6. RCRII-gRNA (BCTV Worland) sequence and map. Supplementary sequence 7. BCTV (Worland) sequence and map. Supplementary sequence 8. MeMV sequence and map. Supplementary sequence 9. Non-specific-sgRNA sequence and map. (PDF 3031 kb) [file 13059_2015_799_MOESM4_ESM.pdf]

SUPPLEMENTARY INFORMATION: ADDITIONAL FILE 4

CRISPR/Cas9-mediated viral interference in plants

Zahir Ali[1], Aala Abulfaraj[1], Ali Idris[1], Shakila Ali[1], Manal Tashkandi[1], and Magdy M. Mahfouz[1, 2]

[1] Laboratory for Genome Engineering, Center for Desert Agriculture & Division of Biological Sciences, 4700 King Abdullah University of Science and Technology, Thuwal 23955-6900, Saudi Arabia

Supplementary sequences and Maps

Supplementary Sequence 1. TYLCV 2.3 genome

GGCCATCCGTATAATATTACCGGATGGCCGCGAATTTTGTGTGGGCCCCTCAACGCACTAA
CTGACAAGGACATGCGAACCAATCAAATTGCATCCTCAAACGTTAGATAAGTGTTCATTTGT
cTTTATATACTTGGTCCCCAAGTATTTTGTCTTGCAATATGTGGGACCCACTTCTAAATGAATT
TCCTGAATCTGTTCACGGATTTCGTTGTATGTTAGCTATTAAATATTTGCAGGCTGTTGAGGA
AACTTACGAGCCCAATACATTGGGCCACGATTTAATTAGGGATCTTATATCTGTTGTAAGGG
CCCGTGACTATGTCGAAGCGACCAGGCGATATAATCATTTCCACGCCCGTCTCGAAGGTTCG
CCGAAGGCTGAACTTCGACAGCCCATACAGCAGCCGTGCTGCTGTCCCCATTGTCCAAGGCA
CAAACAAGCGACGATCATGGACGTACAGGCCCATGTACCGAAAGCCCAGAATATACAGAAT
GTATCGAAGCCCTGATGTTCCCCGTGGATGTGAAGGCCCATGTAAAGTCCAGTCTTATGAGC
AACGGGATGATATTAAGCATACTGGTATTGTTCGTTGTGTTAGTGATGTTACTCGTGGATCTG
GAATTACTCACAGAGTGGGTAAGAGGTTCTGTGTTAAATCGATATATTTTTAGGTAAAGTC
TGGATGGATGAAAATATCAAGAAGCAGAATCACACTAATCAGGTCATGTTCTTCTTGGTCCG
TGATAGAAGGCCTTATGGAAGCAGCCCAATGGATTTTGGACAGGTTTTTAATATGTTCGATA
ATGAGCCCAGTACCGCAACCGTGAAGAATGATTTGCGGGATAGGTTTCAAGTGATAAGGAA
ATTTCATGCTACAGTTATTGGTGGGCCCTCTGGAATGAAGGAACAGGCATTAGTTAAGAGAT
TTTTTAGAATTAACAGTCATGTAACTTATAATCATCAGGAGGCAGCCAAGTATGAGAACCAT
ACTGAGAACGCCTTGTTATTGTATATGGCATGTACGCATGCCTCTAATCCAGTGTATGCAACT
ATGAAAATACGCATCTATTTCTATGATTCAATATCAAATTAATAAATTTTATATTTTATATCA
TGACTTTCTGTTACATTTATTGTGTTTTCAAGTACATCATACAATACATGATCAACTGCTCTG
ATTACATTGTTAATGGAAATTACACCAAGACTATCTAAATACTTAAGAACTTGATATCTAAA
TACTCTTAAGAAACGACCAGTCTGAGGCCGTAAGGTCGTCCAGATTTGGAAGTTGAGATAAC
ATTTGTGAATCCCCAGTACCTTCCTGATATTGTGATTGAATCTTATCTGTATTGAAATGATGT
CGTGGCTCATTAGAAATGGCCTCTCGTCGTGGTTGGTGATCTTGAAATATAGGGGATTTTCTA
TCTCCCATATAAAAACGCCATTCTGGGCTTGATGAGCAGTGATGAGTTCCCCGGTGCGTGAA
TCCATGATTGATGCAGTTGATGTGGAGGTAATATGAGCATCCGCAGTCGAGGTCTATGCGCT
TACGTCTGACTGGCTTAGTCTTCGCTATGCGGTGTTGGATTTTGATTGGCACTTGAGAACAGT
GGCTCGTAGAGGGTGACGAAGGTTGCATTCTTGAGAGCCCAATTTTTCAAGGATATGTTTTT
TTCTTCGTCTAGATATTCCCTATATGATGAGGTAGGTCCTGGATTGCAGAGGAAGATAGTGG
GAATTCCCCCTTTAATTTGAATCGGCTTCCCGTACTTTGTGTTGCTTTGCCAGTCCCTCTGGGC
CCCCATGAATTCCTTGAAGTGCTTTAAATAATGCGGGTctacgtcatcgatgacgttgtaccacgcatcattactgtac
acctttggacttaggtctagatgtccacataaataattatgtgggcctagagacctggcccacattgtcttccccgttctgctatcaccctcgatgacaatacta
ttaggtctCCATGGCCGCGCAGCGGAAGACATGACGTTCTCGGACACCCATACTTCAAGTTCATCT
GGAACTTGATTAAAAGATGAAGATAAAAAGGGAGAAATATAAGGAGCCGGAGGCTCCTGA
AAAATTCTATCTAAATTTGAATTTAAATTATGAAATTGAAGTATAAAGTCTCTAGGAGCTTTC
TCCTTCAGTATATTGAGGGCCTGAGCTTTGGACCCTGAATTGATTGCCTCGGCATATGCGTCG
TTGGCAGATTGGCAACCTCCTCTAGCTGATCGTCCATCGACTTGGAAAACTCCATGATCAAT
GACGTCTCCGTCTTTTTCCATATAGGATTTGACATCGCTTGAACTCTTAGCTCCCTGAATGTT
CGGATGGAAATGTGCTGACCTGGTTGGGGATGTGAGGTCGAAGAATCTGTTGTTTTTGCACT
GGAACTTTCCTTCGAACTGGATGAGCACATGCAAGTGAGGAGTCCCATCTTCATGAAGCTCT
CTGCAGATTCTAATGAATTTTTTGGAAGTGGGTGTTTGTATATTTAATAATTGGGAAAGTGCT
TCCTCTTTAGTTAGAGAGCATTTGGGATAAGTGAGAAAATAATTTTTGGCATTTATTTTAAAC

CGATTGGGGGCTGCCATATTGACTTGGTCAATCGGAGTCTCTCAACTCTTTCTATGTATTGGT
GTATTGGAGTCCTATATATATGGAGACTCCAATGGCATATATGTAAATATTGTACTTTAATTC
AAAATCATCACGCTCCAAAAAGC

IR, CP and RCR-II target sequences are shown in red with yellow highlight.

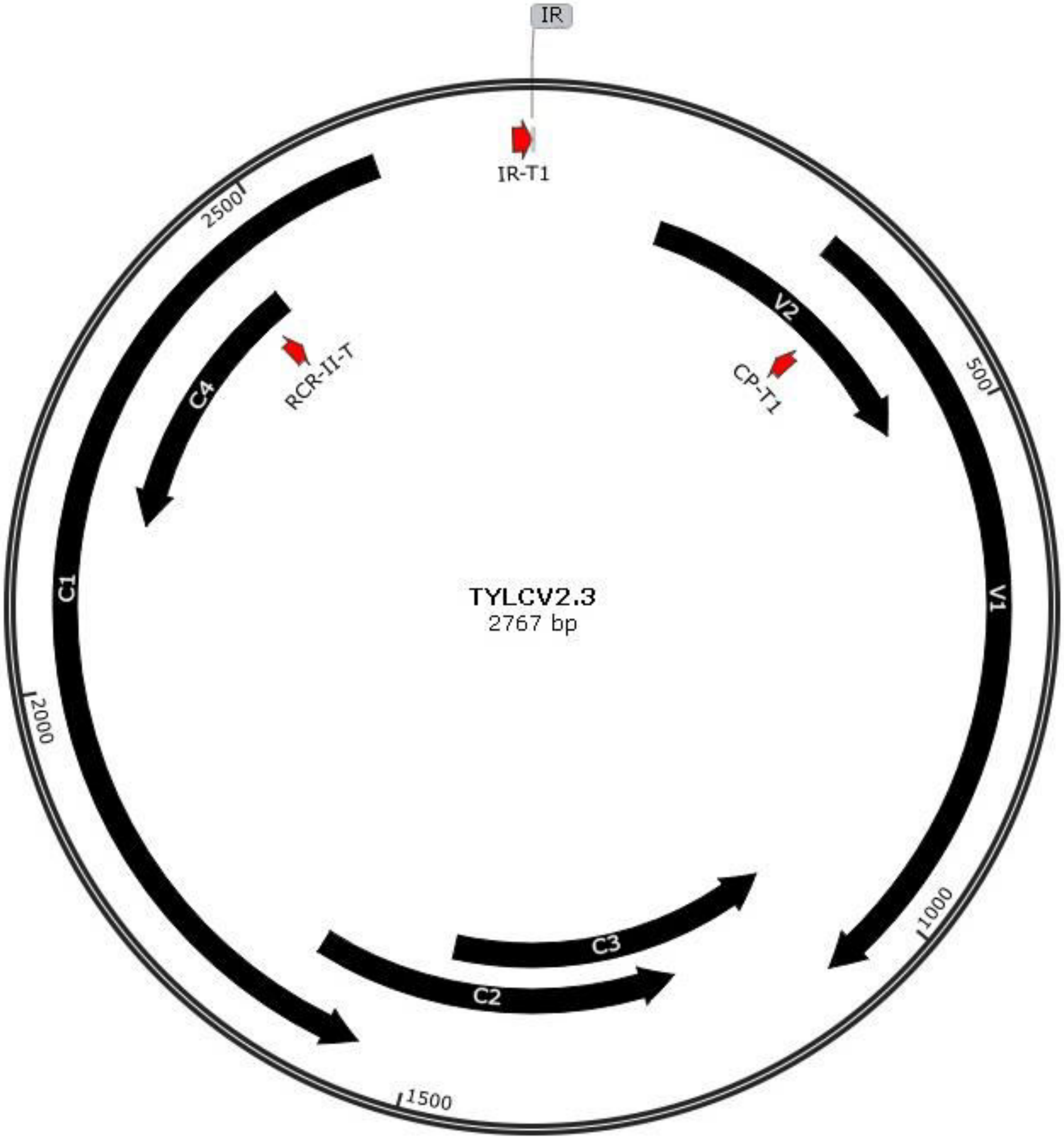

IR, CP and RCR-II Targets are shown as red arrow heads.

Supplementary Sequence 2. IR-gRNA (TYLCV) sequence and map

CgagtctagaGGCCATCCGTATAATATTACgttttagagctagaaatagcaagttaaaataaggctagtccgttatcaactt gaaaaagtggcaccgagtcggtgcttttt

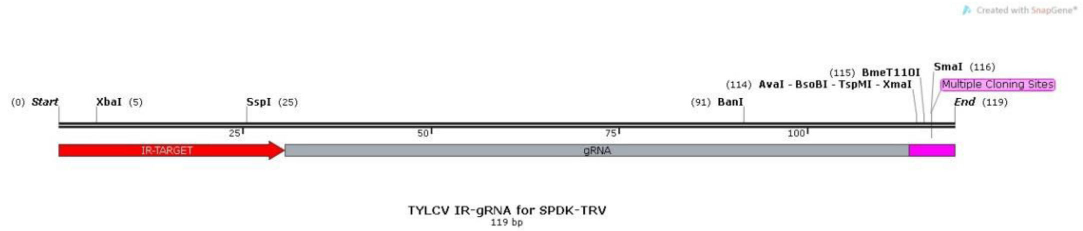

Target sequence is shown in red.

Supplementary Sequence 3. CP-gRNA (TYLCV) sequence and map

CgagtctagaGCTTCGGCGAACCTTCGAGACgttttagagctagaaatagcaagttaaaataaggctagtccgttatcaac ttgaaaaagtggcaccgagtcggtgcttttttcccggg

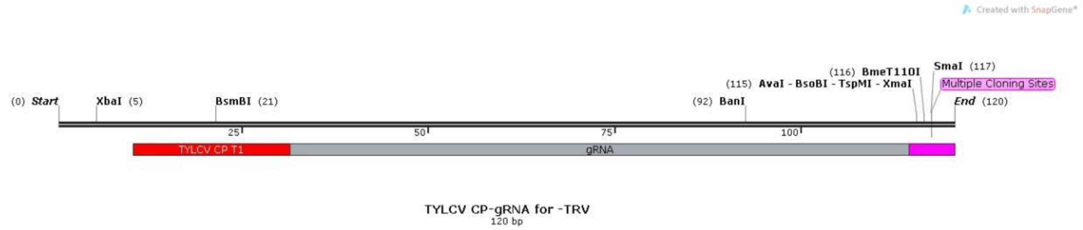

Target sequence is shown in red.

Supplementary Sequence 4. RCRII-gRNA (TYLCV) sequence and map

cgagtctagaGTGGATGAGCACATGCAAGTGGTTTTAGAGCTAGAAATAGCAAGTTAAAATAAGG CTAGTCCGTTATCAACTTGAAAAAGTGGCACCGAGTCGGTGCTTTTTTCCCGGG

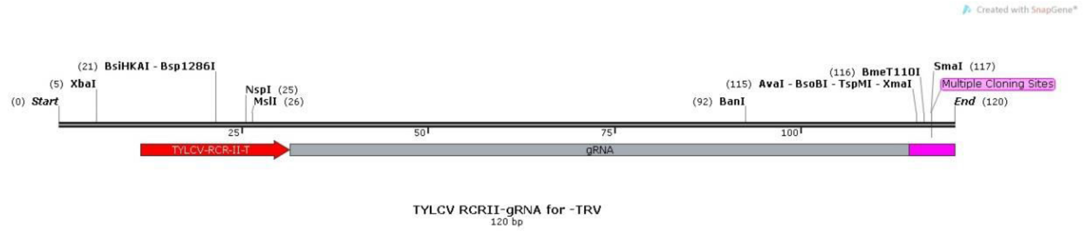

Target sequence is shown in red.

Supplementary Sequence 5. IR-gRNA (BCTV Worland) sequence and map

cgagtctagaGGCCATCCGCAATAATATTACGTTTTAGAGCTAGAAATAGCAAGTTAAAATAAGG
CTAGTCCGTTATCAACTTGAAAAAGTGGCACCGAGTCGGTGCTTTTTTTCCCGGG

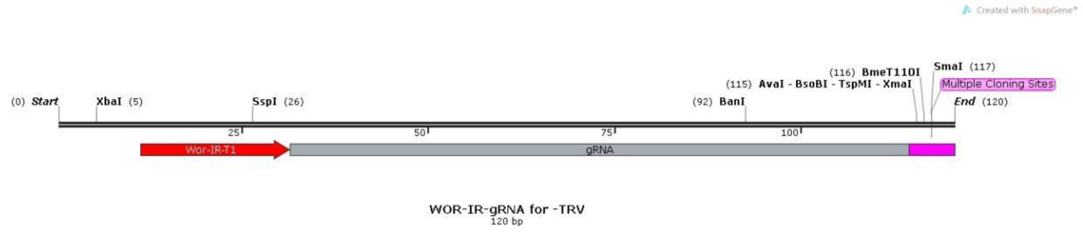

Target sequence is shown in red.

Supplementary Sequence 6. RCRII-gRNA (BCTV Worland) sequence and map

CgagtctagaGCTTTGAATTGGATGAGGGCGGTTTTAGAGCTAGAAATAGCAAGTTAAAATAAGG
CTAGTCCGTTATCAACTTGAAAAAGTGGCACCGAGTCGGTGCTTTTTTTCCCGGG

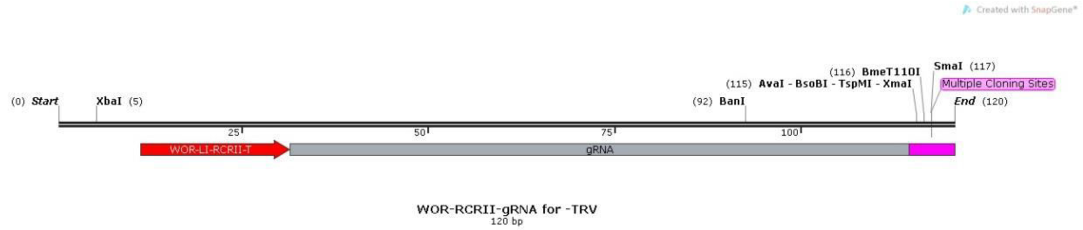

Target sequence is shown in red.

Supplementary Sequence 7. BCTV (Worland) genomic sequence and map.

GCCATCCGCAATAATATTACCGGATGGCCCCGAAAATTTTGCCCCCCCAATTGTAACGTGAC
ACGTGTTATCGCATTATACGTTGTCGGTGCTTGGAAACTTCCTATGTAAGTTTCCTTCTCTAA
CAAGTTTTGACTAGTCAAAAGTTGCGGCCGACAACTACCTTTACTTTTTTAAAGTAAAGTAA
GTTTATTATTCAAAACTTGCTGTGTAAGTTTGGAATATTTTTCTATTTAAATAGCTATACGAA
TACGTATAGGTATTTATAAATGATGGTCTGTTTACCAGACTGGTTATTTTTGCTATTTATCTTC
AGTATTCTACTGCAATCAGGTACGAATTTTTATGGGACCTTTCAGAGTGGATCAATTTCCAG
ACAATTATCCAGCCTTTCTAGCAGTATCGACCAGTTGTTTCTTAAGGTACAACAGGTGGTGT
ATACTAGGTATCCATCAAGAGATAGAGGCTCTGACCCTAGAAGAAGGCGAGGTCTTTCTTCA
ATTCCAGAAGGAAGTGAAGAAGCTACTGAGGTGTAAGGTCAATTTTCATAGGAAGTGTTCGT
TGTATGAGGAAATATACAAGAAATACGTATACAATGTCCCAGAAAAGAAAGGTGAATCCTC
AAAGTGCTTGGCCGAAGAAGAGGAGGACTACTACGACTTCGAGGAAATACCAATGGAGGAG
ACCTGTGACAAAAAACAGGACTCCGAAGTTAAAGATGTATGATGATATGCTTGGTGCTGGTG
GTATAGGATCTACCATTAGTAATAATGGTATGATTACTATGCTGAATAATTATGTCCAGGGT
ATTGGTGATAGTCAGAGAGCGAACAACGTTACTGTGACGAAGCATTTGAAGTTTGATATGGC

TCTTATGGGGAGTTCTCCGTTCTGGGAGACTCCTAATTATATGACCCAATATCATTGGATTAT
CATAGACAAGGATGTTGGGTCAGTGTTCCCTACTAAGTTATCCAGTATATTTGATATTCCGG
ATAACGGTCAGGCTATGCCGTCTACTTATCGTATTCGAAGAGATATGAACGAGAGGTTCATT
GTGAAGAAGAAGTGGAGAACTCATTTGATGTCTACTGGTACTGGCTATGGAGGCAAGGAGA
CTTACAAGGCTCCTTCAATGCCAAATTACAAGAAACCGATGAATATCAATGTCCGTAATCTG
AACATGAGGACTATTTGGAAAGATACCGGAGGTGGGAAGTATGAAGACGTGAAGGAGAAT
GCTTTACTCTATGTTGTTGTTAATGATAATACTGATAATACTAATATGTATGCCACTTTGTTT
GGCAATTGTAGATGTTATTTCTATTAATAAAACTATTTTTTATTTAATACAACTTCATTGCAA
TACTAGTATATTGTATTACACTATTGACGAAATTGAAGACAGAATACAATACATGATTTAAT
ATTTCTATTACAATGTTAATTGAGAGAGAAGATCGTTTTTGATTTCCCTGTCGACTTGGTTC
TTCAAGACTCTCAAAAAACGGTCTCCAGTCAATGTCCTGTGGGATCCAGTTATCGTCATATC
GATCCAACACTTGTGCAGGTTGAGCGATTTGCGGAGGTTGTGGTTGAATCTCATCTGGACTT
TGAGTTGATAAATCGTTCCGTATCTCTCGAACCATAGGAGTTTGAAGTAGAGTGGATTCGGA
ACCGATGTTGTTGGTGTTGATTTCGTCGCCTGTTCCAGGGTAATAGGTAGTTCCGTGCGAAA
ATCCGTGATGGCAGTCATGATGAATTGTGAAGTGACACTTACAGGGGAGATTGACCTTGCGA
GGACGCTTCTGTATCTTTATCAAAGAGAGGGCCGGAGAGTTTAACGAAGGTTGAATTCTGTA
TAGTCCAGGACTTAAGGGCTTCATTTTCTGATTTATCTAGGAAGTCCTGGTAAGAGCTGCCAT
CTCCTGGGTTGCATAATATAATACTGGGAATACCACCTTTAATGACACGTGGTTTTCCATACT
TTAAGTTTGTCTGCCACTCTCTTTGTGCGCCTATGAGGTGTTTCCAATGCTTCATCTTTAAGTA
AGCTGGGTCTACGTCATCAATGACGTTATATAAAACATCATCGTGATATGTTTTTAAACTAA
AATCTAAATGCCCTGAAATGTAATTATGAGGTCCTAATGATCTAGCCCACAAAGTCTTACCC
GTTCTAGAATCACCCTCTATGATTATACTATTATATCTAAAAGGCCGCGCAGCGGCATCCAC
CCCGAAATAAGAATCTGCCCACTCTTGAACAATTTCTGGAACTTGAGTGAAAGAAGATTGTG
GGAATGGAGGTTGATAAATATCTGGTGGAGGAAGAAAAATGGCTTCTAAGTTAGGTTTAAG
GTTGTGATACTGAAAAATATATTTTTCTGGGAGTTTCTCCCTTATTATTTGCAGTGCTTCAGC
TGCATTACCTGCATTTAATGCTTCTGCTGCTGCATCATTAGCCGTCTGCTGGCCTCCTCTAGC
AGATCTTCCGTCGACTTGAAATGTACCCCAGTCGACGTAATCACCGTCCTTCTCGATGTATTG
TTTAACATCGGATGCAGATTTTGCTCCTTGGAAGTTGGGGTGGAAGGTGGAGCTTGAGGAAG
GATGAGTGATGTCGAAGTGTCTAGGGTTTCTGAATTGTGCTTTACCTTTGAATTGGATGAGG
GCGTGGAGATGCAGAGACCCATCCTGATGTTTTTCCTGGGATACTCTAATAAATAATTTATC
AGATGGGCAAGGAATATTTTTCAATATTTCTAGAGCATCTTCTTTTATAACTGAACATCGTGG
GTATGTTAGAAAGATATTTTTGGCTTTAATTTGAAATGATGGTGAACGAGGCATGGTTACTA
TTCCTATTGGGGGCTCTCGTAAACTTGTATGAATGGGGTGCTATGGGAGCCCTTATATACCTC
CAGGG

IR and RCR-II target sequences are shown in red with yellow highlight.

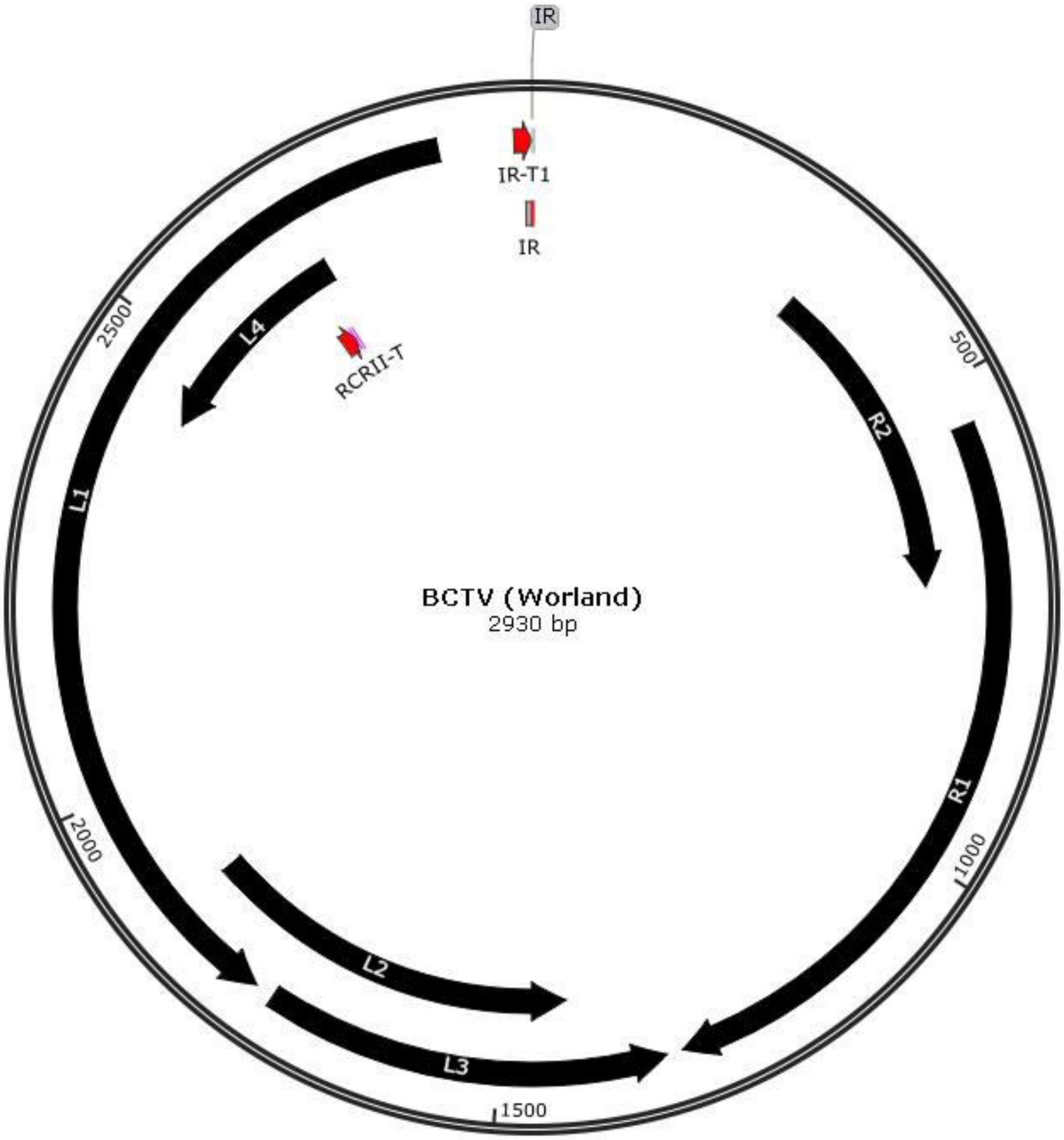

IR and RCR-II Targets are shown as red arrow heads.

Supplementary Sequence 8. MeMV sequence and map

GGCCATCCGCTATAATATTACCGGATGGCCGCCCGCCGCGCCCCCCCTGGGCCCACATATTA
AAGCCGTCCAATCACAAAGCGTCCTGGAAGTCTAATTGTTTAAAATAAGCCTATAAATACAT
TGGAGTCCGTCTATACCCCACCAACTTTAATTTAAAATGGTTAAGAGGGACGCCCCATGGCG
TTTAATGGCGGGGACCACTAAAGTTAGTCGCAACGCCAATTTCTCGCCACGTGGAGGTATGG
GCCCTAAGGCCGCTGCTTGGGTTAACAGGCCCATGTACAGGAAGCCCAGAATTTATCGCACT
TTGAGAGGGCCTGATGTTCCTAAAGGTTGTGAAGGCCCATGTAAGGTACAGTCTTTCGAGCA
GCGTCATGATATTTCTCATGTTGGTAAGGTAATCTGTATATCCGATGTAACTCGTGGTAACGG
TATTACTCACCGTGTTGGCAAGCGTTTTTGTGTGAAGTCTGTGTATATTCTAGGTAAAATATG

GATGGATGAGAACATAAAGCTGAAGAACCACACGAACAGCGTCATGTTTTGGTTGATTCGT
GACAGGAGACCCTATGGTACCCCTATGGATTTTGGTCAGGTGTTTAACATGTATGACAATGA
GCCGAGTACTGCTACCGTCAAGAACGATCTTCGCGATCGATTTCAAGTCATGCATAGGTTCT
ATGCCAAAGTAACTGGTGGTCAGTATGCCAGTAACGAGCAGGCATTGGTTCGGCGATTTTGG
AAGGTTAACAACTACGTCGTGTATAACCATCAGGAAGCAGGAAAATACGAGAATCACACGG
AGAATGCTCTGTTATTGTATATGGCATGTACTCATGCTTCTAATCCTGTGTATGCTACCTTGA
AAATTCGTAGTTATTTTTATGACTCCATTTCGAATTAATAAAGATTAAATTTTATTGAATGTC
TTTCGAGCACACAATTTACATATGGTTTATCCGTTGCGAAACGAACAGCTCTAATGACATTG
TTAAGCGAAACAACACCTAATTGATCTAAATACATTAAAACTAAATCTTTAAATCTATTTAA
ATATGTCGTCCCAGAAGCTTGAACTGATGTCGTCCAGATTTGGAAGTTCAGGTATGCTTTGT
GTAGACTCAACGCCTTCCTCAGGTTGTAGTTGAACCGTATTTGGATGGTGTATATTCTGGTTG
TGGTGTATATTGGCTCCTCCACTTGGATTATCTTGAAATAGAGGGGATTTGGAACCTCCCAG
ATAAAAACGCCATTCATTGCTTGATGAGCAGTGATGGGTTCCCCTGTGCGTGAATCCATGGT
TTCTGCAGTTGATGTGTACGTAAATTGAACAGCCACAGTCCAGGTCTAACCTTCTCCGTCTAA
TGACAGATTTGGATCTCTTCGCTCTCCTGTGCTGTGCTTTGATAGAGGGGGGAGTTGAGGAA
GATGAATTTTGCATTGTGGATCGTCCACGCTCTGAGAGATGCGTTTTCATCTTTATCGAGGAA
GTCTTTATAGCTAGCCCCCTCTCCTGGATTGCACAGCACGATTGAGGGTATTCCTCCTTTAAT
TTGAACTGGCTTCCCGTATTTACAGTTGGACTGCCAGTCCTTTTGGGCCCCTATCAATTCTTT
CCAATGCTTTAATTTCAAATAATTAGGGCTTATGTCATCAATGACGTTATATTCGACGTCATT
CGAATAGACTCTGCCATTAAAGTCAAGATGTCCACTAAGATAATTATGTTTACCTAATGAAC
GGGCCCACATTGTTTTTCCAGTTCGACTATCTCCTTCGATGATGATACTTATCGGTCTATCTG
CCCGCGCAGCGGCACCCCTCCCAAAGTAGTTGTCTGCCCATTCTTGCATCTCATCTGGAACGT
TCGTGAAGGAGGAGAGTTGAAACGGAGGAACCCATGGTTCTGGAGACTTCTGAAATATTCTT
GTTGCGTTCGCAACGAGATTGTGATGTTGAAGAAAGAAGTGTTGTGGTTGTTCCTCCTTTATT
ATTTGCAGTGCTTCCTCTGCAGAAGCTGCGTTTAACGCCTTTGCGTATGTATCGTTAGAAGAC
TGCTGACCTCCTCTAGCAGATCTTCCGTCGATCTGGAATTGTCCCCATTCAATTGTATCTCCG
TCCTTGTCGATGTAGGACTTGACATCGGAGCTGGATTTAGCTCCCTGAATGTTCGGATGGAA
ATGTGCTGACCTGGTTGGGGATACCAAATCGAAGAATCTGTTATTCGTGCAGTTGTATTTTCC
TTCGAACTGGATAAGCACATGGAGATGAGGTTCCCCATTATCGTGAAGCTCTCTACAGATCT
TGATGAATTTTTTGTTTACAGGGGTGTGCAGAGCTTTGATTTGGGACAGTGCTTCTTCTTTGG
CTAATGAACATATAGGGTATGTGAGGAAATAGTTTTTGGCTTTTATTGAGAATGAACCCTTC
CGTGGCATTTTTGTAATAAGGGATGTTCCCCCAATTGCTCCGCTCTCAAAACTCTATATGAAT
CGGGGGAACTGGGGGTACATTTATACTAGAACTCTCATTAAAGGGATTTGCAACACGTGGC

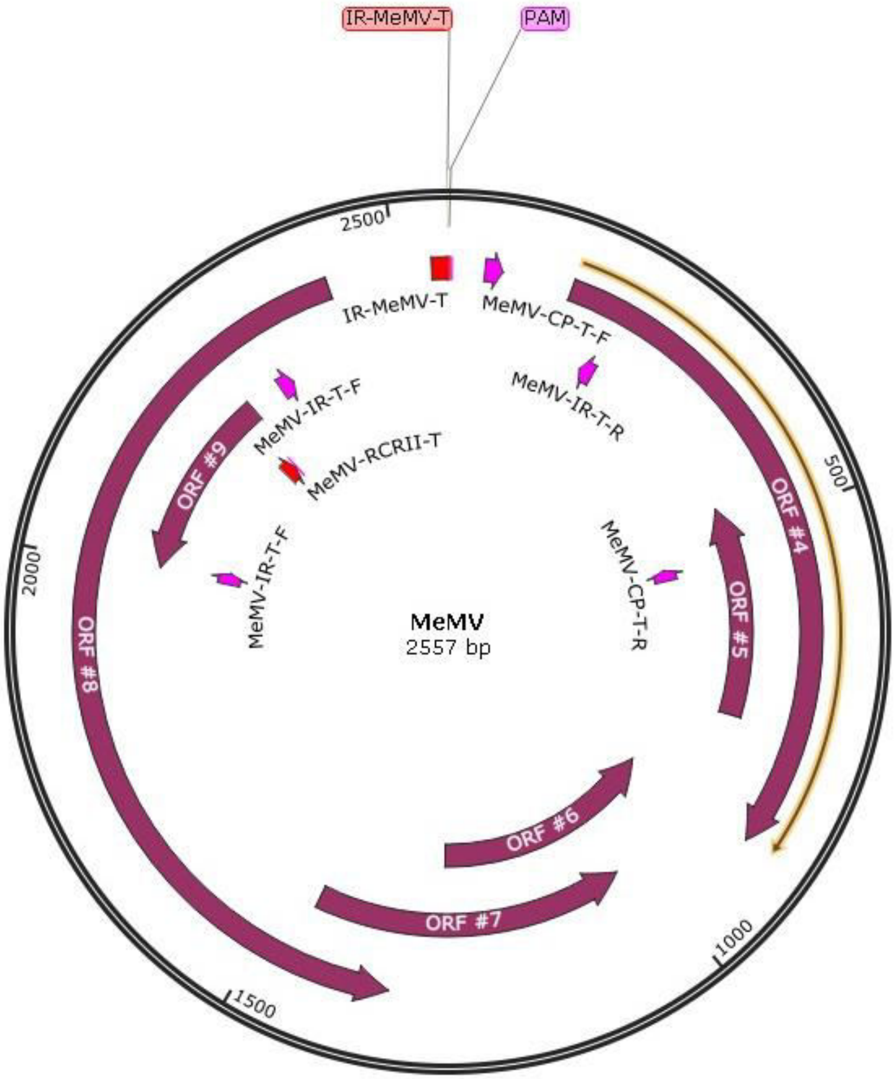

IR Targets is shown in red.

Supplementary Sequence 9. Non-specific sgRNA sequence.
cgagtctaga**GTGGATTCTCTCTGCAAGCCA**GTTTTAGAGCTAGAAATAGCAAGTTAAAATAAG
GCTAGTCCGTTATCAACTTGAAAAAGTGGCACCGAGTCGGTGCTTTTTTTCCCGGG
